# Long‐Term Cardiovascular Risk After Radiotherapy in Women With Breast Cancer

**DOI:** 10.1161/JAHA.117.005633

**Published:** 2017-05-21

**Authors:** Yun‐Jiu Cheng, Xiao‐Ying Nie, Cheng‐Cheng Ji, Xiao‐Xiong Lin, Li‐Juan Liu, Xu‐Miao Chen, Hao Yao, Su‐Hua Wu

**Affiliations:** ^1^ Department of Cardiology The First Affiliated Hospital Sun Yat‐Sen University Guangzhou China; ^2^ Outpatient Department The First Affiliated Hospital Sun Yat‐Sen University Guangzhou China

**Keywords:** cardiotoxicity, cardiovascular complications, cardiovascular disease, cardiovascular disease prevention, cardiovascular disease risk factors, Cardiovascular Disease, Epidemiology, Primary Prevention, Risk Factors, Women

## Abstract

**Background:**

Radiotherapy for breast cancer often involves some incidental exposure of the heart to ionizing radiation. The effect of this exposure on the subsequent risk of heart disease is uncertain. We performed a meta‐analysis to investigate the link between radiotherapy and long‐term cardiovascular morbidity and mortality in patients with breast cancer.

**Methods and Results:**

We performed a literature search using MEDLINE (January 1966 to January 2015) and EMBASE (January 1980 to January 2015) with no restrictions. Studies that reported relative risk (RR) estimates with 95%CIs for the associations of interest were included. Pooled effect estimates were obtained by using random‐effects meta‐analysis. Thirty‐nine studies involving 1 191 371 participants were identified. Patients who received left‐sided radiotherapy, as compared with those receiving right‐sided radiotherapy, experienced increased risks of developing coronary heart disease (RR 1.29, 95%CI 1.13‐1.48), cardiac death (RR 1.22, 95%CI 1.08‐1.37) and death from any cause (RR 1.05, 95%CI 1.01‐1.10). In a comparison of patients with radiotherapy and without radiotherapy, the RRs were 1.30 (95%CI 1.13‐1.49) for coronary heart disease and 1.38 (95%CI 1.18‐1.62) for cardiac mortality. Radiotherapy for breast cancer was associated with an absolute risk increase of 76.4 (95%CI 36.8‐130.5) cases of coronary heart disease and 125.5 (95%CI 98.8‐157.9) cases of cardiac death per 100 000 person‐years. The risk started to increase within the first decade for coronary heart disease and from the second decade for cardiac mortality.

**Conclusions:**

Exposure of the heart to ionizing radiation during radiotherapy for breast cancer increases the subsequent risk of coronary heart disease and cardiac mortality.

**Clinical Perspective**

**What Is New?**

- The association between radiotherapy for breast cancer and long‐term cardiovascular risk remains unclear.
- The meta‐analysis found that, in women diagnosed with breast cancer, risks of coronary heart disease and cardiac mortality were increased in patients with left‐sided radiotherapy compared with those with right‐sided radiotherapy, as well as in the comparison of patients with radiotherapy and those without radiotherapy.

**What Are the Clinical Implications?**

- The meta‐analysis found that, in women diagnosed with breast cancer, radiotherapy increased the risks of coronary heart disease and cardiac mortality.
- Contemporary radiotherapy techniques have likely reduced the risk, but they may not have eliminated cardiotoxicity, and therefore the long‐term hazards in the general population still need to be monitored directly.
- The association between radiotherapy and cardiovascular risk has clinical relevance with respect to individual screening, risk factor modification, and the primary and secondary prevention of heart disease.

## Introduction

Breast cancer is the most common cancer in women, with over a million new cases diagnosed each year worldwide. During the past 30 years, prognosis of breast cancer patients has improved substantially, with 5‐year survival now around 90% in many countries, due partly to earlier diagnosis and greater use of adjuvant therapies.^1^ Although radiotherapy for early breast cancer can reduce the risk of death from the disease several years later, it usually involves some irradiation of the heart, and therefore, much uncertainty still remains regarding the long‐term cardiac effect from breast cancer radiotherapy.^2^

The US SEER (Surveillance Epidemiology and End Results) cancer registries have reported increased risk of cardiovascular death after radiotherapy for breast cancer during the first 2 decades.^3^ A population‐based case‐control study by Darby et al suggested that exposure of the heart to ionizing radiation during breast cancer radiotherapy increased the subsequent rate of ischemic heart disease in a dose‐response relationship.^4^ In contrast, a population‐based cohort study by Boekel et al did not find an increased risk of cardiovascular disease after radiotherapy for ductal carcinoma in situ.^5^ The discordant findings may be explained, in part, by differences in the duration of follow‐up, disease outcomes (eg, coronary heart disease or cardiac death), radiation schemes (eg, older or modern radiotherapy techniques), and radiotherapy fields.

If the relationship between cardiac radiation exposure and the long‐term risk of heart disease were known, then physicians should weigh the likely long‐term benefit of radiotherapy on the breast cancer and the likely long‐term risk of radiation‐induced heart disease and tailor the treatment accordingly. Therefore, we conducted a meta‐analysis with the following aims: (1) to investigate cardiovascular risk in breast cancer patients with left‐sided irradiation or with right‐sided irradiation; (2) to investigate cardiovascular risk in breast cancer patients with or without radiotherapy; (3) to measure the relationship between breast cancer radiotherapy and cardiovascular risk according to different characteristics of the study populations, study designs, follow‐up duration, and treatment eras; and (4) to estimate absolute risk of cardiovascular disease associated with breast cancer radiotherapy in the studies included in the meta‐analysis.

## Methods

### Search Strategy

We followed the PRISMA (Preferred Reporting Items for Systematic reviews and Meta‐Analyses) guidelines for systematic reviews and meta‐analyses.^6^ We searched the publications listed in the electronic databases MEDLINE (source PubMed, January 1, 1966 to January 31, 2014) and EMBASE (January 1, 1980 to January 31, 2014) using the following text and key words in combination both as MeSH terms and text word “radiotherapy OR irradiation OR radiation” AND “breast cancer OR breast tumor” AND “cardiac OR cardiovascular OR heart OR coronary OR toxicity OR morbidity OR mortality OR death.” We searched articles published in any language and scrutinized references from these studies to identify other relevant studies. Ethical approval was not needed.

### Outcomes

The primary study end points were coronary heart disease (defined as diagnosis of ischemic heart disease, acute coronary syndrome, acute myocardial infarction, or death from ischemic heart disease) and cardiac mortality identified using the death registry or validated with medical records. In addition, we included an analysis of death from any cause to examine whether the risk for cardiac death would offset the survival benefit by radiotherapy. We also analyzed the end point of heart failure, arrhythmia, and valvular heart disease that may be associated with cardiovascular toxicity by radiotherapy.

### Study Selection

To minimize differences between studies, we imposed the following methodological restrictions for the inclusion criteria. (1) We used studies that contained the minimum information necessary to estimate the relative risk (RR) associated with radiotherapy and a corresponding measure of uncertainty (ie, 95%CI, standard error, variance, or *P* value of the significance of the estimate). (2) We included cohort studies, case‐control studies, and randomized controlled trials published as original articles; case reports, ecological, and prevalence studies were excluded. (3) We considered studies that were independent. Once full articles were retrieved, studies were further excluded if there was an overlap in patients with another study within the same analysis (in which case, the larger sample size of the 2 studies was selected). If some patients had been included in another study with different analysis (eg, coronary heart disease and cardiac mortality), they would be included once in 1 given analysis. Consequently, there was no overlap in patients included in our meta‐analyses.

### Data Abstraction and Quality Assessment

Two authors (Y.‐J.C. and X.‐Y.N.) extracted the data, and 1 author (C.‐C.J.) independently double‐checked the available data. The following data were extracted from each study: first author's name, publication year, geographical location, mean age, sample size, study design, sampling framework, study population, study end points, number of events, duration of follow‐up, treatment era, and the relative risks and associated measure of variance for all categories of outcomes. When available, we used the most comprehensively adjusted risk estimates. Percentage agreement between the 2 authors on the quality review ranged from 90% to 100%. Any disagreements were resolved by consensus.

To determine the quality of the included studies, we used the Modified Jadad Score for the randomized control trials and the Newcastle‐Ottawa scale for the observational studies. Y.‐J.C. and X.‐Y.N. developed the evaluation criteria (Tables S1 through S3). The score ranged from 0 to 9 points for cohort and case‐control studies and from 0 to 5 points for randomized controlled studies, with a higher score indicating higher study quality.

### Statistical Analysis

The RR was used as a measure of the association between breast cancer radiotherapy and cardiovascular risk. For case‐control studies, the odds ratio was used as an estimate of the RR because cardiovascular events are sufficiently rare.^7^

We assessed the heterogeneity across studies by the Cochran test and by calculating the I^2^ statistic (describing the percentage of total variation across trials that was due to heterogeneity rather than chance), applying the following interpretation for I^2^: <50%=low heterogeneity; 50% to 75%=moderate heterogeneity; >75%=high heterogeneity.^8^, ^9^ We pooled RRs from individual trials according to the method of DerSimonian and Laird for random effects. Subgroup analyses and meta‐regression models were carried out to investigate potential sources of between‐study heterogeneity. We calculated absolute difference in event rate with radiotherapy as {(RR−1)×I_0_}, where RR indicates pooled RRs and I_0_ was the cumulative event rate per 100 000 patient‐years of follow‐up for the reference population. On the basis of population‐based cohort studies, I_0_ was estimated by weighting by the sample size of each study.

Small study bias, consistent with publication bias, was assessed with funnel plot, by the Begg adjusted rank correlation test and by the Egger regression asymmetry test.^8^, ^9^ We also performed the Duval and Tweedie nonparametric “trim and fill” procedure to further assess the possible effect of publication bias in our meta‐analysis.^10^ All *P* values are 2‐sided. Results were considered to be statistically significant at a *P* value of less than 0.05. Statistical analysis was performed with the use of Stata software, version 12.0 (StataCorp, College Station, TX).

## Results

### Study Selection

Our literature search identified 2523 potentially relevant studies as shown in the flow diagram. Of these, 281 articles were considered of interest, and full text was retrieved for detailed evaluation. An additional 242 studies were excluded due to overlap of patients or no relevant outcomes, and finally, 39 studies were included in the meta‐analysis (Figure S1).

### Study Characteristics

A total of 1 191 371 individuals were enrolled in 39 eligible studies, including 11 randomized controlled trials,^11^, ^12^, ^13^, ^14^, ^15^, ^16^, ^17^, ^18^, ^19^, ^20^, ^21^ 13 cancer registry database studies,^5^, ^22^, ^23^, ^24^, ^25^, ^26^, ^27^, ^28^, ^29^, ^30^, ^31^, ^32^, ^33^ and 15 single‐ and multi‐institutional trials.^34^, ^35^, ^36^, ^37^, ^38^, ^39^, ^40^, ^41^, ^42^, ^43^, ^44^, ^45^, ^46^, ^47^, ^48^ Twenty‐two studies were based in Europe, 13 in North America, 1 in Australia and 3 were multinational; 16 studies recruited participants from population registers, and 23 were hospital based (Table S1). The years of patient irradiation ranged from 1949 to 2008, with the study results typically published between 1985 and 2014 and a median follow‐up of 1 to 28 years. Ten trials compared patients who had received radiation therapy with those who had not received radiation therapy; 14 trials compared left‐sided breast cancer radiation therapy and right‐sided breast cancer radiation therapy, and 15 trials compared both. The sizes of the studies ranged from 41 to 558 871, with the 9 largest studies recruiting over 10 000 patients. Of the 28 observational studies, the scores of the Newcastle‐Ottawa scale quality assessment ranged from 5 to 9, and 14 studies had scores of 7 or higher. The methodological quality of the 11 randomized controlled trials was generally good, with Modified Jadad Score of 3 or higher in all the studies (Tables S2 through S4).

### Cardiovascular Morbidity and Mortality in Patients With Left‐Sided Radiotherapy or With Right‐Sided Radiotherapy

#### Coronary Heart Disease

**Figure 1:**
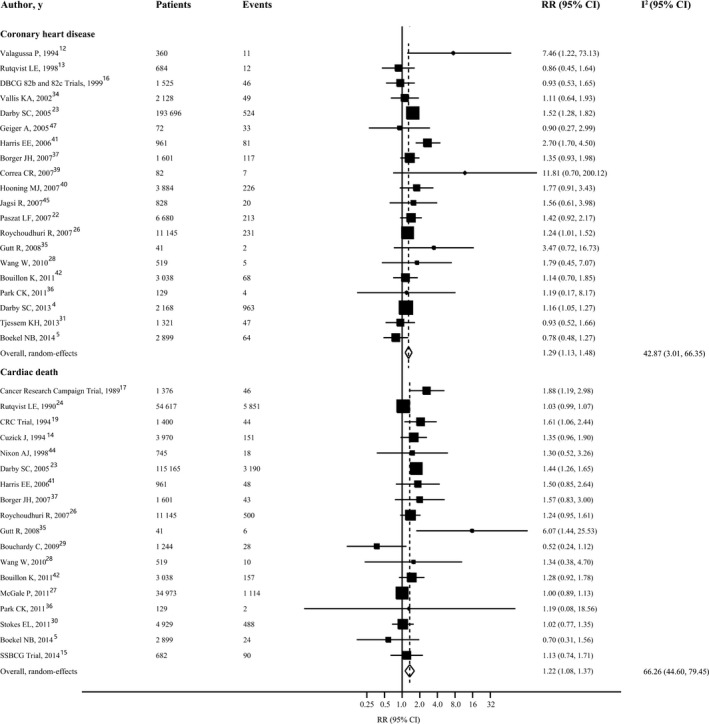
Forest plot for risk of coronary heart disease and cardiac mortality in patients with left‐sided radiotherapy vs those with right‐sided radiotherapy. The size of each square is proportional to the study's weight (inverse of variance). I^2^ indicates variation in RR attributable to heterogeneity; RR, relative risk.

Twenty studies were included for the outcome of coronary heart disease, involving 233 761 participants and 2723 events. In a comparison of women receiving left‐sided radiotherapy with those receiving right‐sided radiotherapy, the overall RR of coronary heart disease was 1.29 (95%CI 1.13‐1.48; *P*<0.001), with low between‐study heterogeneity (I^2^ 42.87%, 95%CI 3.01‐66.35%, *P*=0.02) (Figure 1). Visual inspection of the Begg funnel plot did not identify substantial asymmetry, and the Begg rank correlation test was not statistically significant (*P*=0.23) (Figure S2). However, the Egger linear regression test indicated a possibility of publication bias (*P*=0.02). With the trim‐and‐fill approach, the imputed estimate (RR 1.26, 95%CI 1.09‐1.46, *P*=0.002) was similar to that in the main analysis, indicating that results are unlikely to be explained by publication bias. In a sensitivity analysis that included only the 11 studies that used myocardial infarction or death due to coronary heart disease as the outcome of interest, the pooled RR was 1.34 (95%CI 1.20‐1.50), with no evidence of significant heterogeneity (I^2^ 0%, 95%CI 0‐60.23%, *P*=0.49) (Figure S3).

In the stratified analyses little of the heterogeneity was explained by study type (*P*=0.65), breast cancer staging (*P*=0.69), follow‐up duration (*P*=1.00), age at breast cancer diagnosis (*P*=0.11), number of patients (*P*=0.47), source of patients (*P*=0.83), whether risk profiles were adjusted (*P*=0.83), or whether adjuvant chemotherapy was administered (*P*=0.38). Significant heterogeneity between pooled analyses was noted for studies published before 2010 compared with those published after 2010 (RR 1.42, 95%CI 1.19‐1.69 versus 1.14, 95%CI 1.04‐1.25, *P*=0.03), studies in which cases were validated with medical record compared with studies in which they were not (RR 1.66, 95%CI 1.09‐2.54 versus 1.26, 95%CI 1.10‐1.44, *P*=0.03), and breast cancer diagnosed before 1980 compared with breast cancer diagnosed after 1980 (RR 1.32, 95%CI 1.09‐1.44 versus 1.16, 95%CI 1.01‐1.33, *P*=0.04). The RRs for coronary heart disease in women with left‐sided versus right‐sided radiotherapy, according to the number of years since diagnosis of breast cancer, were as follows: 0 to 4 years, 1.14 (95%CI 0.95‐1.36); 5 to 9 years, 1.17 (95%CI 1.04‐1.32); 10 to 14 years, 1.93 (95%CI 1.13‐3.30); 15 to 20 years, 1.39 (95%CI 1.08‐1.79) (Table 1).

**Table 1:** Stratified Analysis and Heterogeneity Analysis of Relative Risks of Coronary Heart Disease and Cardiac Mortality in Left‐Sided Versus Right‐Sided Radiotherapy

Factors Stratified	Coronary Heart Disease				Cardiac Mortality			
	Patients	Events	RR (95%CI)	*P* Value^a^	Patients	Events	RR (95%CI)	*P* Value^a^
All studies	233 761	2723	1.29 (1.13‐1.48)		239 434	11 810	1.22 (1.08‐1.37)	
Types of studies								
Observational studies	231 876	2666	1.30 (1.13‐1.48)	0.65	232 006	11 479	1.16 (1.01‐1.32)	0.10
RCT	1885	57	2.07 (0.28‐15.05)		7428	331	1.44 (1.18‐1.76)	
Location								
Europe	27 024	1668	1.14 (1.01‐1.29)	0.01	115 344	8005	1.14 (1.01‐1.29)	0.65
North America	10 921	409	1.65 (1.16‐2.34)		6805	562	1.37 (0.88‐2.13)	
Publication year								
<2010	223 687	1572	1.42 (1.19‐1.69)	0.03	192 265	9925	1.34 (1.11‐1.62)	0.08
≧2010	10 074	1151	1.14 (1.04‐1.25)		47 169	1885	1.03 (0.93‐1.14)	
Breast cancer staging								
0 to II	7143	300	1.38 (0.90‐2.11)	0.69	36 152	866	1.40 (1.22‐1.61)	<0.001
I to IV	226 618	2423	1.27 (1.12‐1.43)		131 838	8648	1.07 (0.97‐1.17)	
Surgery								
Breast‐conserving	6130	291	1.80 (1.21‐2.66)	0. 02	98 148	7921	1.12 (1.00‐1.25)	0.58
Mastectomy	7414	331	1.05 (0.77‐1.43)		68 621	2922	1.30 (1.03‐1.64)	
Breast‐conserving or mastectomy	220 217	2101	1.24 (1.09‐1.42)		23 774	1207	1.08 (0.88‐1.33)	
Follow‐up, y								
<20	15 797	483	1.35 (0.93‐1.94)	1.00	47 169	1885	1.18 (0.93‐1.52)	0.52
≧20	217 892	2207	1.28 (1.14‐1.44)		192 265	9925	1.25 (1.08‐1.45)	
Adjusted for risk profiles								
Yes	221 489	1440	1.30 (1.12‐1.53)	0.83	232 387	11 485	1.35 (1.08‐1.69)	0.33
No	12 272	1283	1.36 (1.03‐1.78)		7047	325	1.18 (1.03‐1.35)	
Adjuvant chemotherapy								
Yes	214 908	2237	1.35 (1.12‐1.63)	0.38	159 218	4986	1.13 (0.92‐1.39)	0.30
No	18 853	486	1.20 (1.01‐1.42)		80 216	6824	1.32 (1.08‐1.60)	
Patients, n								
<2000	8123	385	1.42 (1.02‐1.97)	0.47	8698	335	1.41 (1.08‐1.84)	0.11
≧2000	225 638	2338	1.25 (1.10‐1.43)		230 736	11 475	1.15 (1.01‐1.30)	
Age, y								
<55	9659	498	1.23 (1.09‐1.38)	0.11	3307	109	1.49 (1.01‐2.19)	0.21
≧55	224 102	2225	1.60 (1.10‐2.34)		229 381	11 460	1.13 (0.99‐1.28)	
Source of patients								
Population based	227 067	2467	1.28 (1.09‐1.50)	0.83	176 405	5621	1.26 (1.07‐1.48)	0.49
Hospital based	6694	256	1.33 (0.97‐1.83)		63 029	6189	1.16 (0.92‐1.48)	
Case validation								
Yes	6640	1166	1.66 (1.09‐2.54)	0.03	9012	323	1.56 (1.28‐1.91)	0.01
No	227 121	1557	1.26 (1.10‐1.44)		230 422	11 487	1.12 (1.00‐1.27)	
Period of breast cancer diagnosis								
<1980	84 335	5046	1.32 (1.21‐1.44)	0.04	81 730	10 855	1.45 (1.14‐1.89)	0.04
≧1980	88 680	3455	1.16 (1.01‐1.33)		143 606	6680	1.15 (0.92‐1.44)	
Years since breast cancer diagnosis								
1 to 4	308 190	1534	1.14 (0.95‐1.36)	NA	173 683	4794	1.02 (0.96‐1.08)	NA
5 to 9	303 821	1445	1.17 (1.04‐1.32)		166 014	2801	1.05 (0.97‐1.14)	
10 to 14	302 376	1250	1.93 (1.13‐3.30)		164 387	1125	1.26 (1.11‐1.44)	
15 to 20	285 165	736	1.39 (1.08‐1.79)		124 462	929	1.44 (1.14‐1.82)	

RCT indicates randomized controlled trial; RR, relative risk.

a *P*‐values test homogeneity between strata.

In a sensitivity analysis of women who had not been irradiated, the relative risk of coronary heart disease, left versus right tumor laterality, was close to 1 (1.02, 95%CI 0.95‐1.10, *P*=0.60) (Figure S4). Using the average event rate in women receiving right‐sided radiotherapy from included studies and the summary estimates obtained from all studies combined, left‐sided breast cancer radiotherapy was associated with an absolute risk increase of 66.8 (95%CI 37.6‐102.5) cases of coronary heart disease per 100 000 person‐years.

#### Cardiac Mortality

Eighteen studies with data of 239 434 individuals and at least 11 810 events reported risk estimates for cardiac mortality. Overall, compared with women who had received radiotherapy for a right‐sided tumor, women who had received radiotherapy for a left‐sided breast cancer experienced a significantly increased risk for cardiac mortality (RR 1.22, 95%CI 1.08‐1.37, *P*=0.002). There was evidence of moderate heterogeneity of RRs across these studies (I^2^ 66.26%, 95%CI 44.60% to 79.45%, *P<*0.001) (Figure 1). Neither funnel plots nor Egger and Begg tests showed evidence of publication bias (Egger, *P*=0.88; Begg, *P*=0.47) (Figure S2).

To explore the study heterogeneity, we performed analyses subdivided by a number of key study characteristics and clinical factors. Study type, geographical area, surgery type, publication year, follow‐up duration, age at breast cancer diagnosis, number of patients, source of patients, whether risk profiles were adjusted, or whether adjuvant chemotherapy was administered were not significant sources of heterogeneity. However, we found higher risks in studies in which cases were validated with medical records than in studies in which they were not (RR 1.56, 95%CI 1.28‐1.91 versus 1.12, 95%CI 1.00‐1.27, *P*=0.01). In addition, breast cancer diagnosis period also seemed to be associated with the results (RR 1.45, 95%CI 1.14‐1.89 for women diagnosed before 1980 and irradiated versus 1.15, 95%CI 0.92‐1.44 for women diagnosed after 1980 and irradiated; *P*=0.04). The RRs for cardiac mortality, left versus right tumor laterality, increased steeply with time since diagnosis of breast cancer: 0 to 4 years, 1.02 (95%CI 0.96‐1.08); 5 to 9 years, 1.05 (95%CI 0.97‐1.14); 10 to 14 years, 1.26 (95%CI 1.11‐1.44); 15 to 20 years, 1.44 (95%CI 1.14‐1.82) (Table 1).

For women not given radiotherapy, there was little evidence of an association between breast cancer laterality and subsequent cardiac mortality (1.02, 95%CI 0.98‐1.06, *P*=0.28) (Figure S5). Compared with women receiving right‐sided radiotherapy, women with left‐sided radiotherapy experienced an absolute risk increase of 73.9 (95%CI 41.6‐113.9) cases of cardiac death per 100 000 person‐years.

#### Secondary End Points

For death from any cause, 13 studies were included, reporting 96 725 events among 534 419 participants. Left‐sided radiotherapy was associated with a significantly increased risk of death from any cause compared with right‐sided radiotherapy (RR 1.05, 95%CI 1.01‐1.10, *P*=0.02) (Figure S6). We also had additional analysis of other end points in relation to coronary heart disease. Similarly, women with left‐sided radiotherapy, as compared with the reference group, appeared to experience increased risk of death from coronary heart disease (RR 1.23, 95%CI 1.07‐1.41, *P*=0.004), death from myocardial infarction (RR 1.35, 95%CI 1.12‐1.63, *P*=0.002), and myocardial infarction (RR 1.21, 95%CI 1.05‐1.39, *P*=0.007) (Table 2). However, left‐sided radiotherapy did not seem to be associated with risks of heart failure, arrhythmia, and valvular heart disease (Table 2).

**Table 2:** Summary Estimates of Relative Risks of Other Cardiovascular Outcomes Associated With Radiotherapy for Breast Cancer

End Points	No. of Studies	No. of Participants	No. of Events	RR (95%CI)	I^2^ (95%CI) a	*P* Value^b^
Left‐sided radiotherapy vs right‐sided radiotherapy						
Death from any cause	13	534 419	96 725	1.05 (1.01‐1.10)	57.23 (20.69‐76.94)	0.01
Death from coronary heart disease	13	252 654	5103	1.23 (1.07‐1.41)	54.41 (14.75‐75.62)	0.01
Death from myocardial infarction	10	221 515	4476	1.35 (1.12‐1.63)	49.61 (0‐75.59)	0.04
Myocardial infarction	14	126 998	5337	1.21 (1.05‐1.39)	47.75 (2.93‐71.87)	0.02
Heart failure	7	59 157	2570	1.00 (0.91‐1.12)	7.69 (0‐73.05)	0.37
Arrhythmia	7	58 630	2645	1.00 (0.92‐1.07)	0 (0‐70.81)	0.68
Valvular heart disease	4	57 699	640	1.22 (0.91‐1.66)	35.79 (0‐75.96)	0.18
Radiotherapy vs no radiotherapy						
Death from any cause	8	50 346	8388	1.01 (0.85‐1.20)	90.33 (83.35‐94.38)	<0.001
Death from coronary heart disease	8	34 083	1152	1.40 (1.12‐1.75)	60.47 (14.16‐81.80)	0.01
Death from myocardial infarction	4	5628	53	1.84 (0.56‐6.04)	78.93 (43.61‐92.13)	0.003
Myocardial infarction	9	69 358	3278	1.33 (1.002‐1.77)	77.95 (58.27‐88.35)	<0.001
Heart failure	9	70 354	11 235	1.17 (0.90‐1.51)	74.55 (50.71‐86.86)	<0.001
Arrhythmia	3	15 749	252	1.04 (0.69‐1.58)	30.80 (0‐92.80)	0.24
Valvular heart disease	4	20 580	239	1.97 (0.84‐4.62)	61.04 (0‐86.96)	0.05

RR indicates relative risk.

a I^2^ is a measure of the variation in RR attributable to heterogeneity.

b *P* value for I^2^.

### Cardiovascular Morbidity and Mortality in Patients With Radiotherapy or Without Radiotherapy

#### Coronary Heart Disease

**Figure 2:**
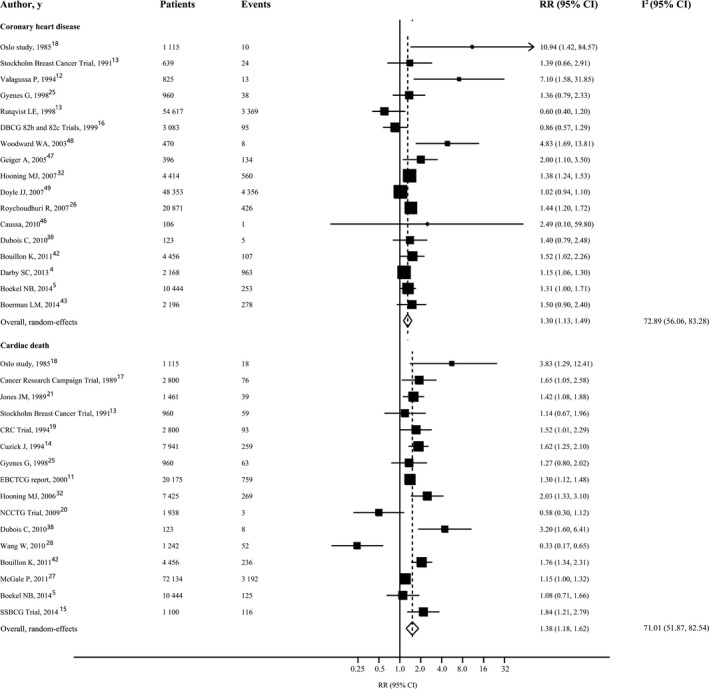
Forest plot for risk of coronary heart disease and cardiac mortality in patients with radiotherapy vs those without radiotherapy. The size of each square is proportional to the study's weight (inverse of variance). I^2^ indicates variation in RR attributable to heterogeneity; RR, relative risk.

From 17 studies analyzed, there were 10 640 cases of coronary heart disease among 155 236 participants. In a comparison of women who received radiotherapy with those who received no radiotherapy, the RR for coronary heart disease was 1.30 (95%CI 1.13‐1.49, *P*<0.001), with moderate heterogeneity (I^2^ 72.89%, 95%CI 56.06% to 83.28%, *P*<0.001) (Figure 2). Visual inspection of the Begg funnel plot revealed asymmetry (*P*<0.001). This raises the possibility of publication bias, although neither the Begg nor the Egger test was statistically significant (Begg, *P*=0.48; Egger, *P*=0.22) (Figure S2). Because of this, we undertook a sensitivity analysis using the trim‐and‐fill method, and the pooled analysis incorporating the hypothetical studies continued to show a statistically significant association between radiotherapy and risk of coronary heart disease (RR 1.18, 95%CI 1.03‐1.36, *P*=0.01). Further analysis of 9 studies that used myocardial infarction or death from coronary heart disease as the end point yielded similar results (RR 1.39, 95%CI 1.10‐1.76, *P*=0.01) (Figure S7).

The finding of increased risk of coronary heart disease in patients who received radiotherapy was consistently found in all of the stratified analyses. Study quality characteristics did not seem to markedly influence the results, although a stronger association was observed for studies in which cases of coronary heart disease were confirmed by laboratory examination or medical record (*P*=0.004). Notably, although treatment era seemed to be associated with the results, patients diagnosed after 1980 with modern radiotherapy techniques still experienced increased risk of coronary heart disease compared with those without radiotherapy (RR 1.21, 95%CI 1.01‐1.37, *P*=0.04). The risk increase started within the first 5 years and continued into the third decade after radiotherapy: the RRs were 1.40 (95%CI 1.12‐1.74) for 0 to 4 years, 1.70 (95%CI 1.18‐2.45) for 5 to 9 years, 1.79 (95%CI 1.08‐2.97) for 10 to 14 years, 1.23 (95%CI 1.02‐1.48) for 15 to 19 years, and 1.88 (95%CI 1.08‐3.27) for 20 years or more (Table 3). From studies that reported information on person‐years in patients with radiotherapy and without radiotherapy, we could calculate that women with radiotherapy experienced an absolute risk increase of 76.4 (95%CI 36.8‐130.5) cases of coronary heart disease per 100 000 person‐years.

**Table 3:** Stratified Analysis and Heterogeneity Analysis of Relative Risks of Cardiac Death and Coronary Heart Disease in Radiotherapy Versus No Radiotherapy

Factors Stratified	Coronary Heart Disease				Cardiac Mortality			
	Patients	Events	RR (95%CI)	*P* Value^a^	Patients	Events	RR (95%CI)	*P* Value^a^
All studies	155 236	10 640	1.30 (1.13‐1.49)		137 074	5367	1.38 (1.18‐1.62)	
Types of studies								
Observational studies	149 104	10 490	1.26 (1.11‐1.44)	0.31	98 245	3948	1.33 (1.01‐1.75)	0.74
RCT	6132	150	2.63 (1.05‐6.58)		38 829	1383	1.43 (1.17‐1.75)	
Location								
Europe	106 017	6142	1.29 (1.13‐1.17)	0.02	113 719	4553	1.53 (1.31‐1.79)	0.01
North America	866	142	2.78 (1.20‐6.40)		3180	55	0.44 (0.25‐0.76)	
Publication year								
<2010	135 743	9033	1.32 (1.06‐1.63)	0.86	47 575	1638	1.43 (1.22‐1.67)	0.68
≧2010	19 493	1607	1.20 (1.10‐1.31)		89 499	3929	1.30 (0.89‐1.88)	
Breast cancer staging								
0 to II	67 136	3670	1.21 (0.69‐2.11)	0.43	41 332	1253	1.36 (1.01‐1.67)	0.95
I to IV	88 100	6970	1.32 (1.14‐1.53)		95 742	4114	1.39 (1.07‐1.82)	
Surgery								
Breast‐conserving	54 617	3369	0.60 (0.35‐1.04)	0.20	···	···	···	
Mastectomy	10 681	735	1.47 (1.02‐2.12)		11 319	472	1.59 (1.31‐1.94)	0.10
Breast‐conserving or mastectomy	89 938	6536	1.31 (1.13‐1.52)		125 755	4895	1.21 (0.97‐1.52)	
Follow‐up, year								
<20	72 870	4071	1.20 (0.88‐1.63)	0.43	21 144	471	1.01 (0.71‐1.45)	0.02
≧20	81 847	6430	1.32 (1.11‐1.57)		115 807	4888	1.51 (1.29‐1.77)	
Adjusted for risk profiles								
Yes	148 875	10 484	1.26 (1.10‐1.44)	0.31	110 750	4688	1.43 (1.22‐1.66)	0.50
No	6361	156	2.10 (1.12‐3.95)		26 324	679	1.28 (0.91‐1.80)	
Adjuvant chemotherapy								
Yes	116 751	9782	1.23 (1.03‐1.46)	0.17	104 137	4399	1.25 (0.93‐1.68)	0.40
No	38 485	858	1.42 (1.24‐1.62)		32 937	968	1.50 (1.33‐1.71)	
Patients, n								
<2000	4634	233	2.04 (1.36‐3.07)	0.10	8899	358	1.26 (0.83‐1.92)	0.47
≧2000	150 602	10 407	1.20 (1.05‐1.37)		128 175	5009	1.43 (1.24‐1.66)	
Age, y								
<55	8011	860	2.00 (1.21‐3.30)	0.06	9363	272	1.11 (0.33‐3.80)	0.81
≧55	144 511	9708	1.19 (1.03‐1.37)		110 634	4551	1.31 (1.04‐1.64)	
Source of patients								
Population based	147 915	10 446	1.25 (1.09‐1.44)	0.34	108 451	4364	1.14 (0.88‐1.48)	0.09
Hospital based	7321	194	1.83 (1.12‐3.06)		28 623	1003	1.56 (1.29‐1.89)	
Case validation								
Yes	5231	449	2.26 (1.43‐3.57)	0.004	24 142	726	1.68 (1.23‐2.27)	0.03
No	150 005	10 191	1.20 (1.05‐1.36)		122 932	4641	1.25 (1.05‐1.50)	
Period of breast cancer diagnosis								
<1980	9913	882	1.52 (1.29‐1.79)	0.02	22 493	843	1.58 (1.39‐1.80)	0.04
≧1980	70 699	6053	1.21 (1.01‐1.37)		33 084	1060	1.27 (0.82‐1.97)	
Years since breast cancer diagnosis								
1 to 4	15 944	537	1.40 (1.12‐1.74)	NA	22 211	212	1.04 (0.51‐2.12)	NA
5 to 9	7596	408	1.70 (1.18‐2.45)		11 672	149	1.17 (0.55‐2.48)	
10 to 14	9066	614	1.79 (1.08‐2.97)		10 332	210	1.41 (1.04‐1.92)	
15 to 19	26 800	844	1.23 (1.02‐1.48)		14 578	375	1.63 (1.21‐2.21)	
>20	4612	259	1.88 (1.08‐3.27)		13 324	1128	1.59 (1.33‐1.91)	

RCT indicates randomized controlled trial; RR, relative risk.

a *P*‐Values test homogeneity between strata.

#### Cardiac Mortality

For the end point of cardiac mortality in women with radiotherapy versus no radiotherapy, 16 studies involving 137 074 patients and 5367 events were included. Compared with women who had not received radiotherapy, those who had received radiotherapy had a 1.38‐fold (95%CI 1.18‐1.62, *P*<0.001) higher risk of cardiac mortality (Figure 2). Although there was moderate heterogeneity among the available studies (I^2^ 71.01%, 95%CI 51.87% to 82.54%, *P*<0.001), the Begg and Egger tests indicated no evidence of publication bias (Begg, *P*=0.96; Egger, *P*=0.12) (Figure S2).

When we evaluated prespecified potential sources of heterogeneity, the major sources were follow‐up duration, case validation, and breast cancer diagnosis period. The RRs were higher in studies with follow‐up duration of more than 20 years than in those with follow‐up duration of less than 20 years (RR 1.51, 95%CI 1.29‐1.77, versus 1.01, 95%CI 0.71‐1.45, *P*=0.02), studies with case validation than those without case validation (RR 1.68, 95%CI 1.23‐2.27, versus 1.25, 95%CI 1.05‐1.50, *P*=0.03), and breast cancer diagnosed before 1980 than that diagnosed after 1980 (RR 1.58, 95%CI 1.39‐1.80, versus 1.27, 95%CI 0.82‐1.97, *P*=0.04). The RRs of cardiac mortality were 1.04 (95%CI 0.51‐2.12) during 0 to 4 years after primary diagnosis, 1.17 (95%CI 0.55‐2.48) during 5 to 9 years, 1.41 (95%CI 1.04‐1.92) during 10 to 14 years, 1.63 (95%CI 1.21‐2.21) during 15 to 19 years, and 1.59 (95%CI 1.33‐1.91) after 20 years (Table 3). The absolute excess risk of cardiac mortality for patients receiving radiotherapy, as compared with those who received no radiotherapy, was 125.5 (95%CI 98.8‐157.9) cases per 100 000 person‐years.

#### Secondary End Points

For the end points associated with coronary heart disease, elevated RRs were observed for death from coronary heart disease (RR 1.40, 95%CI 1.12‐1.75, *P*=0.003) and myocardial infarction (RR 1.33, 95%CI 1.002‐1.77, *P*=0.049). Analysis of 4 studies involving 5628 patients and 53 events showed a statistically nonsignificant 1.84‐fold increased risk for death from myocardial infarction. In addition, we found no significant association between radiotherapy and risk of death from any cause, heart failure, arrhythmia, and valvular heart disease (Table 2).

## Discussion

The present meta‐analysis, involving 1 191 371 patients from 39 studies, has assessed the strength and consistency of associations between breast cancer radiotherapy and subsequent risk of cardiovascular disease. We have shown that in women diagnosed with breast cancer, coronary heart disease and cardiac mortality were increased in the comparison of patients with left‐sided radiotherapy and those with right‐sided radiotherapy, as well as in the comparison of patients with radiotherapy and those without radiotherapy. In subgroup analyses the increase in risk with radiotherapy seemed to be driven by a stronger association among studies in which the cases were validated with medical records and breast cancer was diagnosed before 1980. The increase of risk started within the first decade for coronary heart disease and from the second decade for cardiac mortality and continued into the third decade after radiotherapy.

Previous studies have identified that atomic bomb survivors and patients given radiotherapy for peptic ulcer disease had dose‐related increases in late cardiac morbidity and mortality, whereas conflicting results were reported for patients receiving radiotherapy for breast cancer.^50^, ^51^ Results from our analysis suggest that breast cancer radiotherapy is independently associated with risk of coronary heart disease and cardiac mortality. The risk magnitude appears to be less robust than those reported for well‐established major risk factors such as smoking, hypertension, diabetes mellitus, or hyperlipidemia. It is of note that lack of case validation tends to deflate the pooled risk estimate, indicating that the true magnitude of association between breast cancer radiotherapy and cardiovascular risk may be greater. In absolute terms, 76.4 excess cases of coronary heart disease and 125.5 excess cases of cardiac death occurred per 100 000 person‐years associated with radiotherapy. The absolute risk is not small, and given that postoperative radiotherapy is one of the most commonly used adjuvant therapies and that over a million women develop breast cancer annually, the total number of excess cases of coronary heart disease and cardiac death may not be negligible. In addition, our results showed that radiotherapy did not provide improvement in the overall survival, and even left‐sided radiotherapy could increase risk of death from any cause, indicating that absolute cardiovascular risk can be weighed against the probable absolute reduction in the risk of recurrence or death from breast cancer that would be achieved with radiotherapy. In spite of the clear association between radiotherapy and cardiovascular risk in breast cancer patients, the American College of Cardiology/American Heart Association guidelines do not provide specific recommendations regarding radiation‐induced coronary heart disease or cardiovascular disease.^52^

There is 1 factor that may confound the interpretation of cardiovascular risk associated with radiotherapy in breast cancer patients. Outside the context of randomized trials, patients may have been selected for radiotherapy according to age, nature of breast cancer, and factors associated with the prognosis, and these factors can potentially produce selection biases.^27^ Therefore, simple comparisons of subsequent mortality of irradiated and unirradiated patients might not reliably reflect the real benefit and hazard of treatments. It seems unlikely, however, that the effect can be attributed completely to selection bias because some effect remains even when only coronary heart disease and cardiac mortality are considered, and the effect for heart disease is strong even during the period over 10 years after breast cancer diagnosis. Furthermore, in the randomized trials that involved less selection bias, both risks for coronary heart disease and cardiac mortality were increased in irradiated versus unirradiated patients. But outside the randomized trials, comparisons that avoid selection biases could also be performed, as breast cancer laterality plays little part in determining who should be given radiotherapy, and the cardiac radiation dose is generally greater in left‐sided than in right‐sided breast cancer.^53^, ^54^ Therefore, comparisons of cardiovascular morbidity and mortality in irradiated women with left‐sided versus right‐sided breast cancer can give a valid indication of the extent of any radiation‐related cardiac effect even though the magnitude of risk is likely to be underestimated. The consistency of the findings of increased risk of coronary heart disease and cardiac mortality in patients with left‐sided radiotherapy versus right‐sided radiotherapy as well as in patients with radiotherapy versus those without radiotherapy suggests a causal effect of radiotherapy on heart disease.

Although previous studies have indicated that radiation‐associated cardiovascular complications involved injury of pericardium, myocardium, valves, and conduction systems, our results suggest that radiation‐induced heart disease might be mainly mediated by radiation damage to the coronary arteries leading to myocardial ischemia and myocardial infarction.^55^ First, our study identified that radiotherapy was associated with an increased risk for coronary heart disease but not for heart failure, arrhythmia, and valvular heart disease. The association persists and remains statistically significant across a number of stratified analyses exploring clinical and study quality factors and also persists in the analyses of the outcomes that are less likely to be misclassified such as myocardial infarction and death from coronary heart disease. And death from coronary heart disease may account for the largest number of deaths from cardiac cause, of which the risk is also increased in association with radiotherapy. Second, there is an appropriate temporal relationship: radiotherapy preceded incidence of coronary heart disease in all studies. The risk for coronary heart disease started within the first decade, whereas risk for cardiac death started from the second decade after radiation exposure. This is consistent with existing knowledge that it may take a patient with coronary heart disease a certain period of time to develop into cardiac death. Third, contemporary radiotherapy techniques can involve substantially lower cardiac exposures than those used previously, and therefore, the decline in risk observed for the more recent treatment period provides indirect evidence of a dose‐response relationship for radiation‐induced coronary heart disease. Fourth, there is biological plausibility for causality in that radiation may lead to oxidative stress, inflammatory cell infiltration, endothelial injury, fibrosis of the intima, a prothrombotic state, and atherosclerosis in the coronary arteries and thus may contribute to an increased risk of ischemic heart disease.^55^

Over the past few decades, improvements in radiotherapy planning have reduced cardiac radiation exposures. This situation has been achieved partly by omitting irradiation of the internal mammary chain of lymph nodes, switching from orthovoltage to megavoltage, use of respiratory gating, or blocking the heart in tangential fields.^56^ However, dosimetric studies have demonstrated that modern techniques could not eliminate cardiac exposure, and for approximately half of left‐sided patients, part of the heart still received more than 20 Gy, probably including part of the left anterior descending coronary artery.^57^ This suggests that contemporary radiotherapy techniques may still increase the risk of ischemic heart disease in a proportion of patients, consistent with our findings that risk for coronary heart disease still slightly increased for patients irradiated after 1980. Hardenberg et al also indentified that modern radiotherapy techniques were associated with defects in myocardial perfusion, abnormalities in wall motion, and declines in ejection fraction of the heart.^58^ Notably, the excess risk of cardiac death associated with breast cancer did not become clear until more than 10 years after exposure. Thus, a possible explanation for the lack of any definite hazard of cardiac death from our analysis of a subgroup of patients diagnosed after 1980 is that the follow‐up is not long enough. Therefore, care should continue to be taken to minimize cardiac exposure as much as is practical in patients undergoing radiotherapy for breast cancer.

Strengths of this meta‐analysis include the strict inclusion criteria, the large number of patients analyzed, the robustness of the findings in sensitivity analyses, and the fact that all subgroup analyses were prespecified a priori. There are several limitations to this study. First, there is heterogeneity of RRs among studies in the primary analysis. However, stratified analyses showed pooled RRs consistently greater than 1 across a number of clinical factors. Second, the funnel plot analysis showed some asymmetry, suggesting the possibility of publication bias for coronary heart disease. The trim‐and‐fill sensitivity analysis did not change the general result, suggesting that the association is not an artifact of unpublished negative studies. Third, lack of individual participant data may preclude determining the independent associations of individual variables with study outcomes. Instead, we used between‐study meta‐regressions when possible. Fourth, because radiation doses to the whole heart and to the left anterior descending coronary artery could not be obtained, direct assessment of a dose‐response relationship for radiation‐induced heart disease was not available. We therefore compared cardiovascular risk between different treatment eras as a surrogate, which might to some extent reflect the difference in the dose of radiation to the heart. Fifth, because the individual participant data were not available, we could not calculate the risk for every subcategory of coronary heart disease. We therefore used the composite end point of coronary heart disease instead, which might not be optimal but could be appropriate in assessing the cardiovascular risk associated with breast cancer radiotherapy.^59^, ^60^, ^61^, ^62^ Sixth, given that there are no best tools available for the evaluation of the methodologic quality of randomized controlled trials, we used the most widely used Jadad scoring system as a surrogate.^63^, ^64^, ^65^

In conclusion, the results from this meta‐analysis suggest that radiotherapy for breast cancer might increase the risk of coronary heart disease as well as cardiac mortality. The increase of risk started within the first decade for coronary heart disease and from the second decade for cardiac mortality. Contemporary radiotherapy techniques have likely reduced the risk, but they may not have eliminated cardiotoxicity, and therefore, the long‐term hazards in the general population still need to be monitored directly. The association between radiotherapy and cardiovascular risk has clinical relevance with respect to individual screening, risk factor modification, and the primary and secondary prevention of heart disease.

## Sources of Funding

This work was supported by National Natural Science Foundation of China (No. 81370285), Guangdong Province Science and Technology Program (No. 2012B031800091), and Guangzhou City Science and Technology Program (No. 201508020057) to Dr Wu; and National Natural Science Foundation of China for Young Scholar (No. 81600260) and Natural Science Foundation of Guangdong Province (No. 2016A030313210) to Dr Cheng.

## Disclosures

None.

## Supporting information


**Table S1.** Studies Included in the Meta‐Analysis That Assessed Cardiac Morbidity and Mortality Associated With Breast Cancer Radiotherapy
**Table S2.** The Quality Assessment of Included Cohort Studies Using the Newcastle‐Ottawa Scale
**Table S3.** The Quality Assessment of Included Case‐Control Studies Using the Newcastle‐Ottawa Scale
**Table S4.** The Quality Assessment of Included Randomized Controlled Studies Using the Modified Jadad Scores
**Figure S1.** Flowchart of the selection of studies included in meta‐analysis.
**Figure S2.** Funnel plots showing association of coronary heart disease and cardiac death with radiotherapy.
**Figure S3.** Forest plot for risk of myocardial infarction and death from coronary heart disease in patients with left‐sided radiotherapy versus right‐sided radiotherapy.
**Figure S4.** Forest plot for risk of coronary heart disease in unirradiated patients with left‐sided versus right‐sided breast cancer.
**Figure S5.** Forest plot for risk of cardiac death in unirradiated patients with left‐sided versus right‐sided breast cancer.
**Figure S6.** Forest plot for risk of death from any cause in patients with left‐sided radiotherapy versus right‐sided radiotherapy.
**Figure S7.** Forest plot for risk of myocardial infarction and death from coronary heart disease in patients with radiotherapy versus those without radiotherapy.

## References

[1] DeSantis CE , Lin CC , Mariotto AB , Siegel RL , Stein KD , Kramer JL , Alteri R , Robbins AS , Jemal A . Cancer treatment and survivorship statistics, 2014. CA Cancer J Clin. 2014;64:252–271. 24890451 10.3322/caac.21235

[2] Favourable and unfavourable effects on long‐term survival of radiotherapy for early breast cancer: an overview of the randomised trials. Early Breast Cancer Trialists’ Collaborative Group. Lancet. 2000;355:1757–1770. 10832826

[3] Giordano SH , Kuo YF , Freeman JL , Buchholz TA , Hortobagyi GN , Goodwin JS . Risk of cardiac death after adjuvant radiotherapy for breast cancer. J Natl Cancer Inst. 2005;97:419–424. 15770005 10.1093/jnci/dji067 PMC1853253

[4] Darby SC , Ewertz M , McGale P , Bennet AM , Blom‐Goldman U , Bronnum D , Correa C , Cutter D , Gagliardi G , Gigante B , Jensen MB , Nisbet A , Peto R , Rahimi K , Taylor C , Hall P . Risk of ischemic heart disease in women after radiotherapy for breast cancer. N Engl J Med. 2013;368:987–998. 23484825 10.1056/NEJMoa1209825

[5] Boekel NB , Schaapveld M , Gietema JA , Rutgers EJ , Versteegh MI , Visser O , Aleman BM , van Leeuwen FE . Cardiovascular morbidity and mortality after treatment for ductal carcinoma in situ of the breast. J Natl Cancer Inst. 2014;106:dju156. 25128694 10.1093/jnci/dju156 PMC4151854

[6] Stroup DF , Berlin JA , Morton SC , Olkin I , Williamson GD , Rennie D , Moher D , Becker BJ , Sipe TA , Thacker SB . Meta‐analysis of observational studies in epidemiology: a proposal for reporting. Meta‐analysis Of Observational Studies in Epidemiology (MOOSE) group. JAMA. 2000;283:2008–2012. 10789670 10.1001/jama.283.15.2008

[7] Cheng YJ , Liu ZH , Yao FJ , Zeng WT , Zheng DD , Dong YG , Wu SH . Current and former smoking and risk for venous thromboembolism: a systematic review and meta‐analysis. PLoS Med. 2013;10:e1001515. 24068896 10.1371/journal.pmed.1001515 PMC3775725

[8] Cheng YJ , Yao FJ , Liu LJ , Tang K , Lin XX , Li WJ , Zhang J , Wu SH . B‐type natriuretic peptide and prognosis of end‐stage renal disease: a meta‐analysis. PLoS One. 2013;8:e79302. 24236118 10.1371/journal.pone.0079302 PMC3827377

[9] Egger M , Davey SG , Schneider M , Minder C . Bias in meta‐analysis detected by a simple, graphical test. BMJ. 1997;315:629–634. 9310563 10.1136/bmj.315.7109.629 PMC2127453

[10] Higgins JP , Thompson SG . Quantifying heterogeneity in a meta‐analysis. Stat Med. 2002;21:1539–1558. 12111919 10.1002/sim.1186

[11] Clarke M , Collins R , Darby S , Davies C , Elphinstone P , Evans E , Godwin J , Gray R , Hicks C , James S , MacKinnon E , McGale P , McHugh T , Peto R , Taylor C , Wang Y . Effects of radiotherapy and of differences in the extent of surgery for early breast cancer on local recurrence and 15‐year survival: an overview of the randomised trials. Lancet. 2005;366:2087–2106. 16360786 10.1016/S0140-6736(05)67887-7

[12] Valagussa P , Zambetti M , Biasi S , Moliterni A , Zucali R , Bonadonna G . Cardiac effects following adjuvant chemotherapy and breast irradiation in operable breast cancer. Ann Oncol. 1994;5:209–216. 8186169 10.1093/oxfordjournals.annonc.a058795

[13] Rutqvist LE , Lax I , Fornander T , Johansson H . Cardiovascular mortality in a randomized trial of adjuvant radiation therapy versus surgery alone in primary breast cancer. Int J Radiat Oncol Biol Phys. 1992;22:887–896. 1555981 10.1016/0360-3016(92)90784-f

[14] Cuzick J , Stewart H , Rutqvist L , Houghton J , Edwards R , Redmond C , Peto R , Baum M , Fisher B , Host H . Cause‐specific mortality in long‐term survivors of breast cancer who participated in trials of radiotherapy. J Clin Oncol. 1994;12:447–453. 8120544 10.1200/JCO.1994.12.3.447

[15] Killander F , Anderson H , Kjellen E , Malmstrom P . Increased cardio and cerebrovascular mortality in breast cancer patients treated with postmastectomy radiotherapy—25 year follow‐up of a randomised trial from the South Sweden Breast Cancer Group. Eur J Cancer. 2014;50:2201–2210. 24951164 10.1016/j.ejca.2014.04.033

[16] Hojris I , Overgaard M , Christensen JJ , Overgaard J . Morbidity and mortality of ischaemic heart disease in high‐risk breast‐cancer patients after adjuvant postmastectomy systemic treatment with or without radiotherapy: analysis of DBCG 82b and 82c randomised trials. Radiotherapy Committee of the Danish Breast Cancer Cooperative Group. Lancet. 1999;354:1425–1430. 10543669 10.1016/s0140-6736(99)02245-x

[17] Haybittle JL , Brinkley D , Houghton J , A'Hern RP , Baum M . Postoperative radiotherapy and late mortality: evidence from the Cancer Research Campaign trial for early breast cancer. BMJ. 1989;298:1611–1614. 2503148 10.1136/bmj.298.6688.1611 PMC1836871

[18] Host H , Brennhovd IO , Loeb M . Postoperative radiotherapy in breast cancer—long‐term results from the Oslo study. Int J Radiat Oncol Biol Phys. 1986;12:727–732. 3519550 10.1016/0360-3016(86)90029-5

[19] Houghton J , Baum M , Haybittle JL . Role of radiotherapy following total mastectomy in patients with early breast cancer. The Closed Trials Working Party of the CRC Breast Cancer Trials Group. World J Surg. 1994;18:117–122. 8197766 10.1007/BF00348201

[20] Halyard MY , Pisansky TM , Dueck AC , Suman V , Pierce L , Solin L , Marks L , Davidson N , Martino S , Kaufman P , Kutteh L , Dakhil SR , Perez EA . Radiotherapy and adjuvant trastuzumab in operable breast cancer: tolerability and adverse event data from the NCCTG Phase III Trial N9831. J Clin Oncol. 2009;27:2638–2644. 19349549 10.1200/JCO.2008.17.9549 PMC2690390

[21] Jones JM , Ribeiro GG . Mortality patterns over 34 years of breast cancer patients in a clinical trial of post‐operative radiotherapy. Clin Radiol. 1989;40:204–208. 2647360 10.1016/s0009-9260(89)80099-6

[22] Paszat LF , Mackillop WJ , Groome PA , Schulze K , Holowaty E . Mortality from myocardial infarction following postlumpectomy radiotherapy for breast cancer: a population‐based study in Ontario, Canada. Int J Radiat Oncol Biol Phys. 1999;43:755–762. 10098430 10.1016/s0360-3016(98)00412-x

[23] Darby SC , McGale P , Taylor CW , Peto R . Long‐term mortality from heart disease and lung cancer after radiotherapy for early breast cancer: prospective cohort study of about 300,000 women in US SEER cancer registries. Lancet Oncol. 2005;6:557–565. 16054566 10.1016/S1470-2045(05)70251-5

[24] Rutqvist LE , Johansson H . Mortality by laterality of the primary tumour among 55,000 breast cancer patients from the Swedish Cancer Registry. Br J Cancer. 1990;61:866–868. 2372488 10.1038/bjc.1990.193 PMC1971705

[25] Gyenes G , Rutqvist LE , Liedberg A , Fornander T . Long‐term cardiac morbidity and mortality in a randomized trial of pre‐ and postoperative radiation therapy versus surgery alone in primary breast cancer. Radiother Oncol. 1998;48:185–190. 9783890 10.1016/s0167-8140(98)00062-0

[26] Roychoudhuri R , Robinson D , Putcha V , Cuzick J , Darby S , Moller H . Increased cardiovascular mortality more than fifteen years after radiotherapy for breast cancer: a population‐based study. BMC Cancer. 2007;7:9. 17224064 10.1186/1471-2407-7-9 PMC1784099

[27] McGale P , Darby SC , Hall P , Adolfsson J , Bengtsson NO , Bennet AM , Fornander T , Gigante B , Jensen MB , Peto R , Rahimi K , Taylor CW , Ewertz M . Incidence of heart disease in 35,000 women treated with radiotherapy for breast cancer in Denmark and Sweden. Radiother Oncol. 2011;100:167–175. 21752480 10.1016/j.radonc.2011.06.016

[28] Wang W , O'Connell D , Stuart K , Boyages J . Analysis of 10‐year cause‐specific mortality of patients with breast cancer treated in New South Wales in 1995. J Med Imaging Radiat Oncol. 2011;55:516–525. 22008173 10.1111/j.1754-9485.2011.02304.x

[29] Bouchardy C , Rapiti E , Usel M , Majno SB , Vlastos G , Benhamou S , Miralbell R , Neyroud‐Caspar I , Verkooijen HM , Vinh‐Hung V . Excess of cardiovascular mortality among node‐negative breast cancer patients irradiated for inner‐quadrant tumors. Ann Oncol. 2010;21:459–465. 19703922 10.1093/annonc/mdp341

[30] Stokes EL , Tyldesley S , Woods R , Wai E , Olivotto IA . Effect of nodal irradiation and fraction size on cardiac and cerebrovascular mortality in women with breast cancer treated with local and locoregional radiotherapy. Int J Radiat Oncol Biol Phys. 2011;80:403–409. 20584587 10.1016/j.ijrobp.2010.02.041

[31] Tjessem KH , Johansen S , Malinen E , Reinertsen KV , Danielsen T , Fossa SD , Fossa A . Long‐term cardiac mortality after hypofractionated radiation therapy in breast cancer. Int J Radiat Oncol Biol Phys. 2013;87:337–343. 23886416 10.1016/j.ijrobp.2013.05.038

[32] Hooning MJ , Botma A , Aleman BM , Baaijens MH , Bartelink H , Klijn JG , Taylor CW , van Leeuwen FE . Long‐term risk of cardiovascular disease in 10‐year survivors of breast cancer. J Natl Cancer Inst. 2007;99:365–375. 17341728 10.1093/jnci/djk064

[33] Rutter CE , Chagpar AB , Evans SB . Breast cancer laterality does not influence survival in a large modern cohort: implications for radiation‐related cardiac mortality. Int J Radiat Oncol Biol Phys. 2014;90:329–334. 25304793 10.1016/j.ijrobp.2014.06.030

[34] Vallis KA , Pintilie M , Chong N , Holowaty E , Douglas PS , Kirkbride P , Wielgosz A . Assessment of coronary heart disease morbidity and mortality after radiation therapy for early breast cancer. J Clin Oncol. 2002;20:1036–1042. 11844827 10.1200/JCO.2002.20.4.1036

[35] Gutt R , Correa CR , Hwang WT , Solin LJ , Litt HI , Ferrari VA , Harris EE . Cardiac morbidity and mortality after breast conservation treatment in patients with early‐stage breast cancer and preexisting cardiac disease. Clin Breast Cancer. 2008;8:443–448. 18952559 10.3816/CBC.2008.n.054

[36] Park CK , Li X , Starr J , Harris EE . Cardiac morbidity and mortality in women with ductal carcinoma in situ of the breast treated with breast conservation therapy. Breast J. 2011;17:470–476. 21762242 10.1111/j.1524-4741.2011.01122.x

[37] Borger JH , Hooning MJ , Boersma LJ , Snijders‐Keilholz A , Aleman BM , Lintzen E , van Brussel S , van der Toorn PP , Alwhouhayb M , van Leeuwen FE . Cardiotoxic effects of tangential breast irradiation in early breast cancer patients: the role of irradiated heart volume. Int J Radiat Oncol Biol Phys. 2007;69:1131–1138. 17606332 10.1016/j.ijrobp.2007.04.042

[38] Dubois CL , Pappas C , Belmans A , Erven K , Adriaenssens T , Sinnaeve P , Coosemans M , Kayaert P , Weltens C , Desmet W . Clinical outcome of coronary stenting after thoracic radiotherapy: a case‐control study. Heart. 2010;96:678–682. 20424148 10.1136/hrt.2009.183129

[39] Correa CR , Litt HI , Hwang WT , Ferrari VA , Solin LJ , Harris EE . Coronary artery findings after left‐sided compared with right‐sided radiation treatment for early‐stage breast cancer. J Clin Oncol. 2007;25:3031–3037. 17634481 10.1200/JCO.2006.08.6595

[40] Hooning MJ , Aleman BM , van Rosmalen AJ , Kuenen MA , Klijn JG , van Leeuwen FE . Cause‐specific mortality in long‐term survivors of breast cancer: a 25‐year follow‐up study. Int J Radiat Oncol Biol Phys. 2006;64:1081–1091. 16446057 10.1016/j.ijrobp.2005.10.022

[41] Harris EE , Correa C , Hwang WT , Liao J , Litt HI , Ferrari VA , Solin LJ . Late cardiac mortality and morbidity in early‐stage breast cancer patients after breast‐conservation treatment. J Clin Oncol. 2006;24:4100–4106. 16908933 10.1200/JCO.2005.05.1037

[42] Bouillon K , Haddy N , Delaloge S , Garbay JR , Garsi JP , Brindel P , Mousannif A , Le MG , Labbe M , Arriagada R , Jougla E , Chavaudra J , Diallo I , Rubino C , de Vathaire F . Long‐term cardiovascular mortality after radiotherapy for breast cancer. J Am Coll Cardiol. 2011;57:445–452. 21251585 10.1016/j.jacc.2010.08.638

[43] Boerman LM , Berendsen AJ , van der Meer P , Maduro JH , Berger MY , de Bock GH . Long‐term follow‐up for cardiovascular disease after chemotherapy and/or radiotherapy for breast cancer in an unselected population. Support Care Cancer. 2014;22:1949–1958. 24584711 10.1007/s00520-014-2156-9

[44] Nixon AJ , Manola J , Gelman R , Bornstein B , Abner A , Hetelekidis S , Recht A , Harris JR . No long‐term increase in cardiac‐related mortality after breast‐conserving surgery and radiation therapy using modern techniques. J Clin Oncol. 1998;16:1374–1379. 9552040 10.1200/JCO.1998.16.4.1374

[45] Jagsi R , Griffith KA , Koelling T , Roberts R , Pierce LJ . Rates of myocardial infarction and coronary artery disease and risk factors in patients treated with radiation therapy for early‐stage breast cancer. Cancer. 2007;109:650–657. 17238178 10.1002/cncr.22452

[46] Caussa L , Kirova YM , Gault N , Pierga JY , Savignoni A , Campana F , Dendale R , Fourquet A , Bollet MA . The acute skin and heart toxicity of a concurrent association of trastuzumab and locoregional breast radiotherapy including internal mammary chain: a single‐institution study. Eur J Cancer. 2011;47:65–73. 20843680 10.1016/j.ejca.2010.08.013

[47] Geiger AM , Chen W , Bernstein L . Myocardial infarction risk and tamoxifen therapy for breast cancer. Br J Cancer. 2005;92:1614–1620. 15841078 10.1038/sj.bjc.6602562 PMC2362055

[48] Woodward WA , Strom EA , McNeese MD , Perkins GH , Outlaw EL , Hortobagyi GN , Buzdar AU , Buchholz TA . Cardiovascular death and second non‐breast cancer malignancy after postmastectomy radiation and doxorubicin‐based chemotherapy. Int J Radiat Oncol Biol Phys. 2003;57:327–335. 12957242 10.1016/s0360-3016(03)00594-7

[49] Doyle JJ , Neugut AI , Jacobson JS , Wang J , McBride R , Grann A , Grann VR , Hershman D . Radiation therapy, cardiac risk factors, and cardiac toxicity in early‐stage breast cancer patients. Int J Radiat Oncol Biol Phys. 2007;68:82–93. 17336464 10.1016/j.ijrobp.2006.12.019

[50] Preston DL , Shimizu Y , Pierce DA , Suyama A , Mabuchi K . Studies of mortality of atomic bomb survivors. Report 13: solid cancer and noncancer disease mortality: 1950–1997. 2003. Radiat Res. 2012;178:V146–V172. 10.1667/rrav12.1 22870966

[51] Carr ZA , Land CE , Kleinerman RA , Weinstock RW , Stovall M , Griem ML , Mabuchi K . Coronary heart disease after radiotherapy for peptic ulcer disease. Int J Radiat Oncol Biol Phys. 2005;61:842–850. 15708264 10.1016/j.ijrobp.2004.07.708

[52] Fihn SD , Blankenship JC , Alexander KP , Bittl JA , Byrne JG , Fletcher BJ , Fonarow GC , Lange RA , Levine GN , Maddox TM , Naidu SS , Ohman EM , Smith PK . 2014 ACC/AHA/AATS/PCNA/SCAI/STS focused update of the guideline for the diagnosis and management of patients with stable ischemic heart disease: a report of the American College of Cardiology/American Heart Association Task Force on Practice Guidelines, and the American Association for Thoracic Surgery, Preventive Cardiovascular Nurses Association, Society for Cardiovascular Angiography and Interventions, and Society of Thoracic Surgeons. Circulation. 2014;130:1749–1767. 25070666 10.1161/CIR.0000000000000095

[53] Taylor CW , Nisbet A , McGale P , Darby SC . Cardiac exposures in breast cancer radiotherapy: 1950s–1990s. Int J Radiat Oncol Biol Phys. 2007;69:1484–1495. 18035211 10.1016/j.ijrobp.2007.05.034

[54] Vandenbroucke JP . When are observational studies as credible as randomised trials? Lancet. 2004;363:1728–1731. 15158638 10.1016/S0140-6736(04)16261-2

[55] Lee MS , Finch W , Mahmud E . Cardiovascular complications of radiotherapy. Am J Cardiol. 2013;112:1688–1696. 24012026 10.1016/j.amjcard.2013.07.031

[56] Senkus‐Konefka E , Jassem J . Cardiovascular effects of breast cancer radiotherapy. Cancer Treat Rev. 2007;33:578–593. 17764850 10.1016/j.ctrv.2007.07.011

[57] Taylor CW , Povall JM , McGale P , Nisbet A , Dodwell D , Smith JT , Darby SC . Cardiac dose from tangential breast cancer radiotherapy in the year 2006. Int J Radiat Oncol Biol Phys. 2008;72:501–507. 18374500 10.1016/j.ijrobp.2007.12.058

[58] Hardenbergh PH , Munley MT , Bentel GC , Kedem R , Borges‐Neto S , Hollis D , Prosnitz LR , Marks LB . Cardiac perfusion changes in patients treated for breast cancer with radiation therapy and doxorubicin: preliminary results. Int J Radiat Oncol Biol Phys. 2001;49:1023–1028. 11240243 10.1016/s0360-3016(00)01531-5

[59] Ding M , Bhupathiraju SN , Satija A , van Dam RM , Hu FB . Long‐term coffee consumption and risk of cardiovascular disease: a systematic review and a dose‐response meta‐analysis of prospective cohort studies. Circulation. 2014;129:643–659. 24201300 10.1161/CIRCULATIONAHA.113.005925 PMC3945962

[60] Cheng YJ , Li ZY , Yao FJ , Xu XJ , Ji CC , Chen XM , Liu LJ , Lin XX , Yao H , Wu SH . Early repolarization is associated with significantly increased risk for ventricular arrhythmias and sudden cardiac death in patients with structural heart diseases. Heart Rhythm. 2017 Available at: http://www.heartrhythmjournal.com/article/S1547-5271(17)30450-2/fulltext. Accessed May 16, 2017. 10.1016/j.hrthm.2017.04.022 28416467

[61] Cheng YJ , Lin XX , Ji CC , Chen XM , Liu LJ , Tang K , Wu SH . Role of early repolarization pattern in increasing risk of death. J Am Heart Assoc. 2016;5:e003375 DOI: 10.1161/JAHA.116.003375. 27671315 10.1161/JAHA.116.003375 PMC5079012

[62] Cheng YJ , Mei WY , Chen XM , Liu LJ , Zheng DD , Ji CC , Tang K , Wu SH . Long‐term prognosis associated with early repolarisation pattern in Chinese population with atherosclerotic risk factors. Heart. 2016 Available at: http://heart.bmj.com/content/early/2016/12/30/heartjnl-2016-310259.long. Accessed May 16, 2017. 10.1136/heartjnl-2016-310259 28039169

[63] Huwiler‐Muntener K , Juni P , Junker C , Egger M . Quality of reporting of randomized trials as a measure of methodologic quality. JAMA. 2002;287:2801–2804. 12038917 10.1001/jama.287.21.2801

[64] Cheng YJ , Nie XY , Chen XM , Lin XX , Tang K , Zeng WT , Mei WY , Liu LJ , Long M , Yao FJ , Liu J , Liao XX , Du ZM , Dong YG , Ma H , Xiao HP , Wu SH . The role of macrolide antibiotics in increasing cardiovascular risk. J Am Coll Cardiol. 2015;66:2173–2184. 26564594 10.1016/j.jacc.2015.09.029

[65] Bellemain‐Appaix A , O'Connor SA , Silvain J , Cucherat M , Beygui F , Barthelemy O , Collet JP , Jacq L , Bernasconi F , Montalescot G . Association of clopidogrel pretreatment with mortality, cardiovascular events, and major bleeding among patients undergoing percutaneous coronary intervention: a systematic review and meta‐analysis. JAMA. 2012;308:2507–2516. 23287889 10.1001/jama.2012.50788

