# Supplementary material for: Long‐Term Cardiovascular Risk After Radiotherapy in Women With Breast Cancer
Source: J Am Heart Assoc. 2017 May 21;6(5):e005633. doi: 10.1161/JAHA.117.005633 (PMC5524103; doi:10.1161/JAHA.117.005633)
Supplement: Supplementary file 1 — Table S1. Studies Included in the Meta‐Analysis That Assessed Cardiac Morbidity and Mortality Associated With Breast Cancer Radiotherapy Table S2. The Quality Assessment of Included Cohort Studies Using the Newcastle‐Ottawa Scale Table S3. The Quality Assessment of Included Case‐Control Studies Using the Newcastle‐Ottawa Scale Table S4. The Quality Assessment of Included Randomized Controlled Studies Using the Modified Jadad Scores Figure S1. Flowchart of the selection of studies included in meta‐analysis. Figure S2. Funnel plots showing association of coronary heart disease and cardiac death with radiotherapy. Figure S3. Forest plot for risk of myocardial infarction and death from coronary heart disease in patients with left‐sided radiotherapy versus right‐sided radiotherapy. Figure S4. Forest plot for risk of coronary heart disease in unirradiated patients with left‐sided versus right‐sided breast cancer. Figure S5. Forest plot for risk of cardiac death in unirradiated patients with left‐sided versus right‐sided breast cancer. Figure S6. Forest plot for risk of death from any cause in patients with left‐sided radiotherapy versus right‐sided radiotherapy. Figure S7. Forest plot for risk of myocardial infarction and death from coronary heart disease in patients with radiotherapy versus those without radiotherapy. [file JAH3-6-e005633-s001.pdf]

Supplemental Material

Table S1. Studies included in the meta-analysis that assessed cardiac morbidity and mortality associated with breast cancer radiotherapy.

Study	Country	Treatment period	Study type	Study design	End points	Source of participants
**Prospective randomized controlled trial**						
EBCTCG report[1]	Multi-national	1985-2000	Randomized controlled trial	Radiotherapy vs. without radiotherapy	Death from any cause; cardiac mortality	Population-based
Valagussa P[2]	Italy	1980-1990	Randomized controlled trial	Left-sided vs. right-sided radiotherapy; radiotherapy vs. without radiotherapy	Coronary heart disease; arrhythmia	Hospital-based
Woodward WA[3]	USA	1975-1994	Randomized controlled trials	Radiotherapy vs. without radiotherapy	Death from myocardial infarction	Hospital-based
Stockholm Breast Cancer Trial[4]	Sweden	1971-1976	Randomized controlled trial	Radiotherapy vs. without radiotherapy	Death from any cause; cardiac death; death from coronary heart disease	Hospital-based
Cuzick J[5]	UK	1949-1974	Randomized controlled trial	Left-sided vs. right-sided radiotherapy; radiotherapy vs. without radiotherapy	Cardiac mortality	Hospital-based
SSBCG Trial[6]	Sweden	1978-1985	Randomized controlled trial	Left-sided vs. right-sided radiotherapy; Radiotherapy vs. without radiotherapy	Cardiac mortality	Hospital-based
DBCG 82b and 82c	Denmark	1982-1990	Randomized controlled	Left-sided vs. right-sided	Death from any cause;	Hospital-based

Trials[7]			trial	radiotherapy; radiotherapy vs. without radiotherapy	death from coronary heart disease; death from myocardial infarction; coronary heart disease; myocardial infarction	
Cancer Research Campaign Trial[8]	UK	1970-1980	Randomized controlled trial	Left-sided vs. right-sided radiotherapy; radiotherapy vs. without radiotherapy	Death from any cause; cardiac mortality	Hospital-based
Oslo study[9]	Norway	1964-1972	Randomized controlled trial	Radiotherapy vs. without radiotherapy	Cardiac mortality; death form myocardial infarction	Hospital-based
CRC Trial[10]	UK	1970-1975	Randomized controlled trial	Left-sided vs. right-sided radiotherapy; radiotherapy vs. without radiotherapy	Death from any cause; cardiac mortality	Hospital-based
Jones JM[11]	UK	1949-1955	Prospective cohort	Radiotherapy vs. without radiotherapy	Cardiac mortality	Hospital-based
NCCTG Trial[12]	USA	2000-2005	Randomized controlled trial	Radiotherapy vs. without radiotherapy	Cardiac mortality; chronic heart failure	Hospital-based
**Population-based tumor registry/database reviews**						
**Ontario cancer registry**						
Paszat LF[13]	Canada	1973-1992	Prospective cohort	Left-sided vs. right-sided	Death from	Population-based

				radiotherapy	myocardial infarction	
**SEER database cohort study**						
Patt DA[14]	Multi-national	1986-1993	Prospective cohort	Left-sided vs. right-sided radiotherapy	Coronary heart disease; chronic heart failure; valvular heart disease; arrhythmia	Population-based
Pinder MC[15]	Multi-national	1992-2002	Prospective cohort	Left-sided vs. right-sided radiotherapy; Radiotherapy vs. without radiotherapy	Chronic heart failure	Population-based
Harlan LC[16]	USA	1996-1999	Prospective cohort	Radiotherapy vs. without radiotherapy	Chronic heart failure; myocardial infarction	Population-based
Darby SC[17]	Multi-national	1973-2001	Prospective cohort	Left-sided vs. right-sided radiotherapy	Death from any cause; cardiac mortality; death from coronary heart disease; death from myocardial infarction	Population-based
Henson KE[18]	Multi-national	1973-2008	Prospective cohort	Left-sided vs. right-sided radiotherapy	Cardiac mortality	Population-based
Jacobson JS[19]	Multi-national	1982-1990	Prospective cohort	Left-sided vs. right-sided radiotherapy; radiotherapy vs. without radiotherapy	Coronary heart disease; myocardial infarction	Population-based
Giordano SH[20]	USA	1973-2000	Prospective cohort	Left-sided vs. right-sided radiotherapy	Death from coronary heart disease	Population-based
**Swedish Cancer**						

**Registry**						
Rutqvist LE[21]	Sweden	1970-1986	Prospective cohort	Left-sided vs. right-sided radiotherapy	Death from any cause; cardiac mortality; death from myocardial infarction	Population-based
**Stockholm Breast Cancer Study Group**						
Gyenes G[22]	Sweden	1971-1976	Prospective cohort	Radiotherapy vs. without radiotherapy	Cardiac mortality; death from coronary heart disease; death from myocardial infarction; myocardial infarction	Hospital-based
Rutqvist LE[23]	Sweden	1976-1987	Prospective cohort	Left-sided vs. right-sided radiotherapy; radiotherapy vs. without radiotherapy	Myocardial infarction; death from myocardial infarction	Population-based
**Thames Cancer Registry**						
Roychoudhuri R[24]	UK	1971-1998	Prospective cohort	Left-sided vs. right-sided radiotherapy; radiotherapy vs. without radiotherapy	Death from any cause; cardiac mortality; death from coronary heart disease	Population-based
**Swedish National Cancer Registry and DBCCG**						
McGale P[25]	Denmark and	1976-2006	Prospective cohort	Left-sided vs. right-sided	Cardiac mortality;	Population-based

	Sweden			radiotherapy	death from coronary heart disease; death from myocardial infarction; coronary heart disease; myocardial infarction; chronic heart failure; valvular heart disease; arrhythmia	
Darby SC[26]	Denmark and Sweden	1958-2001	Case-control	Left-sided vs. right-sided radiotherapy; radiotherapy vs. without radiotherapy	Coronary heart disease; death from coronary heart disease	Population-based
**NSW Central Cancer Registry**						
Wang W[27]	Australia	1995	Prospective cohort	Left-sided vs. right-sided vs. without radiotherapy	Death from any cause; cardiac mortality; death from coronary heart disease; death from valvular heart disease; death from chronic heart failure	Hospital-based
**Geneva Cancer Registry**						
Bouchardy C[28]	Switzerland	1980-2004	Prospective cohort	Left-sided vs. right-sided radiotherapy	Death from any cause; cardiac mortality	Population-based
**BCCA study**						

Stokes EL[29]	Canada	1990-1996	Retrospective cohort	Left-sided vs. right-sided radiotherapy	Cardiac mortality	Population-based
**NRH radiation therapy registry**						
Tjessem KH[30]	Norway	1975-1991	Prospective cohort	Left-sided vs. right-sided radiotherapy	Death from coronary heart disease	Population-based
**Dutch Late Effects Breast Cancer Cohort**						
Hooning MJ[31]	Netherlands	1970-1986	Prospective cohort	Left-sided vs. right-sided radiotherapy; radiotherapy vs. without radiotherapy	Coronary heart disease; myocardial infarction; chronic heart failure; valvular heart disease	Population-based
**Dutch population-based DCIS cohort**						
Boekel NB[32]	Netherlands	1989-2004	Prospective cohort	Left-sided vs. right-sided radiotherapy; radiotherapy vs. without radiotherapy	Cardiac mortality; coronary heart disease; myocardial infarction; chronic heart failure arrhythmia; valvular heart disease	Population-based
**National Cancer Database**						
Rutter CE[33]	USA	1998-2006	Prospective cohort	Left-sided vs. right-sided	Death from any cause	Population-based

				radiotherapy		
**Single and Multi-institutional reviews**						
Vallis KA[34]	USA	1982-1988	Retrospective cohort	Left-sided vs. right-sided radiotherapy	Death from any cause; death from myocardial infarction; myocardial infarction	Hospital-based
Gutt R[35]	USA	1980-1994	Retrospective cohort	Left-sided vs. right-sided radiotherapy	Cardiac mortality; death from coronary heart disease; death from chronic heart failure	Hospital-based
Park CK[36]	USA	1986-1992	Retrospective cohort	Left-sided vs. right-sided radiotherapy	Death from any cause; cardiac mortality; Coronary heart disease; chronic heart failure; arrhythmia	Hospital-based
Borger JH[37]	Multi-national	1980-1993	Prospective cohort	Left-sided vs. right-sided radiotherapy	Death from any cause; cardiac mortality; death from coronary heart disease; death from myocardial infarction; coronary heart disease	Hospital-based
Dubois C[38]	Belgium	1998-2005	Case-control	Radiotherapy vs. without	Cardiac mortality;	Hospital-based

				radiotherapy	myocardial infarction	
Correa CR[39]	USA	1977-1995	Prospective cohort	Left-sided vs. right-sided radiotherapy	Coronary heart disease	Hospital-based
Hooning MJ[40]	Netherlands	1970-1975	Prospective cohort	Radiotherapy vs. without radiotherapy	Cardiac mortality	Hospital-based
Harris EE[41]	USA	1997-1994	Retrospective cohort	Left-sided vs. right-sided radiotherapy	Death from any cause; cardiac mortality; death from myocardial infarction; death from chronic heart failure; coronary heart disease; myocardial infarction; chronic heart failure; valvular heart disease; arrhythmia	Population-based
Bouillon K[42]	Sweden	1954-1984	Retrospective cohort	Left-sided vs. right-sided radiotherapy; radiotherapy vs. without radiotherapy	Cardiac mortality; death from coronary heart disease; death from chronic heart failure; death from valvular heart disease; arrhythmia	Population-based
Boerman LM[43]	Netherlands	1970-2006	Case-control	Radiotherapy vs. without radiotherapy	Coronary heart disease	Population-based
Nixon AJ [44]	USA	1968-1986	Prospective cohort	Left-sided vs. right-sided radiotherapy	Death from any cause; cardiac mortality	Hospital-based

Jagsi R[45]	USA	1984-2000	Prospective cohort	Left-sided vs. right-sided radiotherapy	Coronary heart disease; myocardial infarction	Hospital-based
Caussa L[46]	France	2003-2007	Prospective cohort	Radiotherapy vs. without radiotherapy	Coronary heart disease	Hospital-based
Geiger A[47]	USA	1980-2000	Case-control	Left-sided vs. right-sided radiotherapy; radiotherapy vs. without radiotherapy	Myocardial infarction	Population-based

Table S2. The quality assessment of included cohort studies using the Newcastle-Ottawa scale.

	**Selection**				**Comparability**		**Outcome**			
Author	Representativeness of Exposed Cohort	Selection of Non-Exposed Cohort	Ascertainment Of Exposure	Demonstration That Outcome of Interest Was Not Present at Start of Study	Adjust for age	Adjust for other factors such as tumor size, tumor grade, type of surgery	Assessment of outcome	Follow-up length	Loss to follow-up rate	Total Quality Score
Wang W, 2011[27]	1	1	1	1	0	0	1	1	0	6
Vallis KA, 2002[34]	0	1	1	0	0	0	1	1	1	5
Rutter CE, 2014[33]	1	1	1	1	1	1	1	1	0	8
Gutt R, 2008[35]	0	1	1	1	0	0	1	1	0	5
Park CK,2011[36]	0	1	1	1	0	0	0	1	1	5
Darby SC, 2005[17]	1	1	1	1	0	0	1	1	1	7
Borger JH, 2007[37]	0	1	1	0	0	0	1	1	1	5
Correa CR,	0	1	1	1	0	0	1	1	1	6

2007[39]										
Hooning MJ, 2006[40]	0	1	1	0	1	0	1	1	1	6
Boekel NB, 2014[32]	1	1	1	1	1	0	1	1	1	8
Bouchardy C, 2008[28]	1	1	1	1	1	1	1	1	0	8
Stokes EL, 2011[29]	1	1	1	1	1	1	1	1	0	8
McGale P, 2011[25]	1	1	1	1	0	0	1	1	0	6
Roychoudhuri R, 2007[24]	1	1	1	1	1	0	1	1	1	8
Harris EE, 2006[41]	1	1	1	1	1	1	1	1	1	9
Gyenes G, 1998[22]	0	1	1	1	1	0	1	1	1	8
Tjessem KH, 2013[30]	1	1	1	1	0	0	1	1	0	6
Bouillon K, 2011[42]	1	1	1	1	1	1	1	1	0	8
Hooning MJ, 2007[31]	0	1	1	0	1	0	1	1	1	6
Rutqvist LE, 1990[21]	1	1	1	1	0	0	1	1	1	7

Paszat LF, 1999[13]	1	1	1	1	1	1	1	1	0	8
Nixon AJ, 1998[44]	0	1	1	1	1	1	1	1	1	9
Jagsi R, 2006[45]	0	1	1	0	1	0	1	1	1	7
Woodward WA, 2003[3]	0	1	1	0	0	0	1	1	1	5
Caussa L, 2010[46]	0	1	1	1	0	0	1	1	1	6

The quality of included studies was assessed by the Newcastle Ottawa scale. A study can be awarded a maximum of one star for each numbered item within the Selection and Outcome categories and a maximum of two stars for Comparability.

Selection: 1) Representativeness of exposed cohort: 1, study population truly or somewhat representative of a community/ population based study; 0, study population was sampled from a special population, ie. population from a company, hospital patients, data from the health insurance company or health examination organization, nurses, Adventist group.

2) Selection of non-exposed cohort: 1, drawn from the same community as the exposed cohort.

3) Ascertainment of exposure: 1, specific dietary assessment method of radiotherapy with validation; 0, no specific dietary assessment method or specific radiotherapy assessment method without validation

4) Demonstration that outcome was not present at start of study: 1, yes; 0, no.

Comparability: 1) 1, whether a study adjusted for age deliberately; 1, whether a study adjusted for other factors such as tumor size, tumor grade, type of surgery

Outcome: 1) Assessment of outcome:1, cases were confirmed by medical records or record linkage; 0, self-reported.

2) Was follow-up long enough for outcomes to occur: 1, duration of follow-up >= 5 year; 0, if duration of follow-up < 5 year.

3) Loss to follow-up rate: 1, complete follow-up or loss to follow up rate <=20 %; 0, follow-up rate < 80% or no description of those lost.

Table S3. The quality assessment of included case-control studies using the Newcastle-Ottawa scale.

	Selection				Comparability		Outcome			
Author	Adequacy of case definition	Representativeness of the cases	Selection of Controls	Definition of Controls	Adjust for age	Adjust for other factors such as tumor size, tumor grade, type of surgery	Assessment of outcome	Same method of ascertainment for cases and controls	Non-Response rate	Total Quality Score
Dubois C, 2010[38]	1	1	0	1	0	0	1	1	0	5
Boerman LM, 2014[43]	1	1	1	1	1	1	1	1	0	8
Geiger A, 2005[47]	1	1	1	1	1	1	1	1	0	8

The quality of included studies was assessed by the Newcastle Ottawa scale. A study can be awarded a maximum of one star for each numbered item within the Selection and Outcome categories and a maximum of two stars for Comparability.

Selection: 1) Adequacy of case definition: 1, cases were confirmed by medical records or record linkage; 0, self-reported.

2) Representativeness of the cases: 1, consecutive or obviously representative series of cases; 0, potential for selection biases or not stated.

3) Selection of Controls: 1, community controls; 0, hospital controls or no description.

4) Definition of Controls: 1, no history of cardiovascular events; 0, no description of source.

Comparability: 1) 1, whether a study adjusted for age deliberately; 1, whether a study adjusted for other factors such as tumor size, tumor grade, type of surgery.

Outcome: 1) Assessment of outcome: 1, cardiovascular events were confirmed by medical records or record linkage; 0, self-reported.

2) Same method of ascertainment for cases and controls: 1, yes; 0, no.

3) Non-Response rate: 1, same rate for both groups; 0, non respondents described rate different and no designation.

Table S4. The quality assessment of included randomized controlled studies using the Modified Jadad Scores.

Author	Randomization	Concealment of allocation	Withdrawals and dropouts	Total
EBCTCG report[1]	2	2	1	5
Valagussa P[2]	1	2	1	4
Stockholm Breast Cancer Trial[4]	1	2	0	3
Cuzick J[5]	1	1	1	3
SSBCG Trial[6]	2	1	1	4
DBCG 82b and 82c Trials[7]	2	2	1	5
Cancer Research Campaign Trial[8]	1	1	1	3
Oslo study[9]	2	1	1	4
CRC Trial[10]	1	1	1	3
NCCTG Trial[12]	1	2	1	4
Jones JM[11]	1	1	1	3

Randomization: 0, not randomized or inappropriate method of randomization; 1, the study was described as randomized; 2, the method of randomization was described and it was appropriate.
Concealment of allocation: 0, not describe the method of allocation concealment; 1, the study was described as using allocation concealment method; 2, the method of allocation concealment was described appropriately.
Double blinding: 0, no blind or inappropriate method of blinding; 1, the study was described as double blind; 2, the method of double blinding was described and it was appropriate.

Withdrawals and dropouts: 0, not describe the follow-up; 1, a description of withdrawals and dropouts.

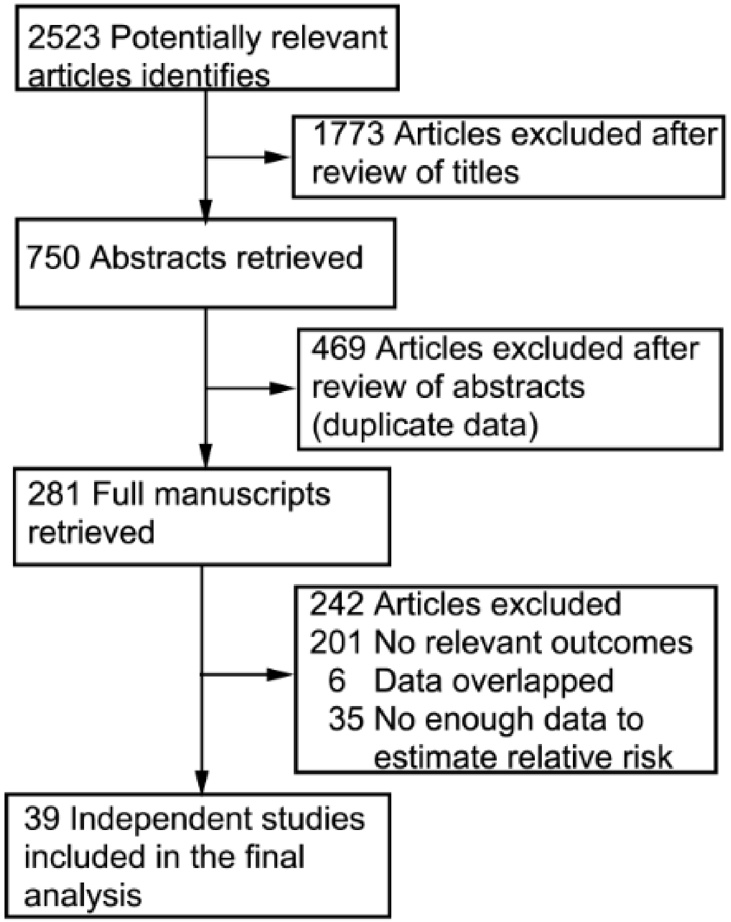

Figure S1. Flowchart of the selection of studies included in meta-analysis.

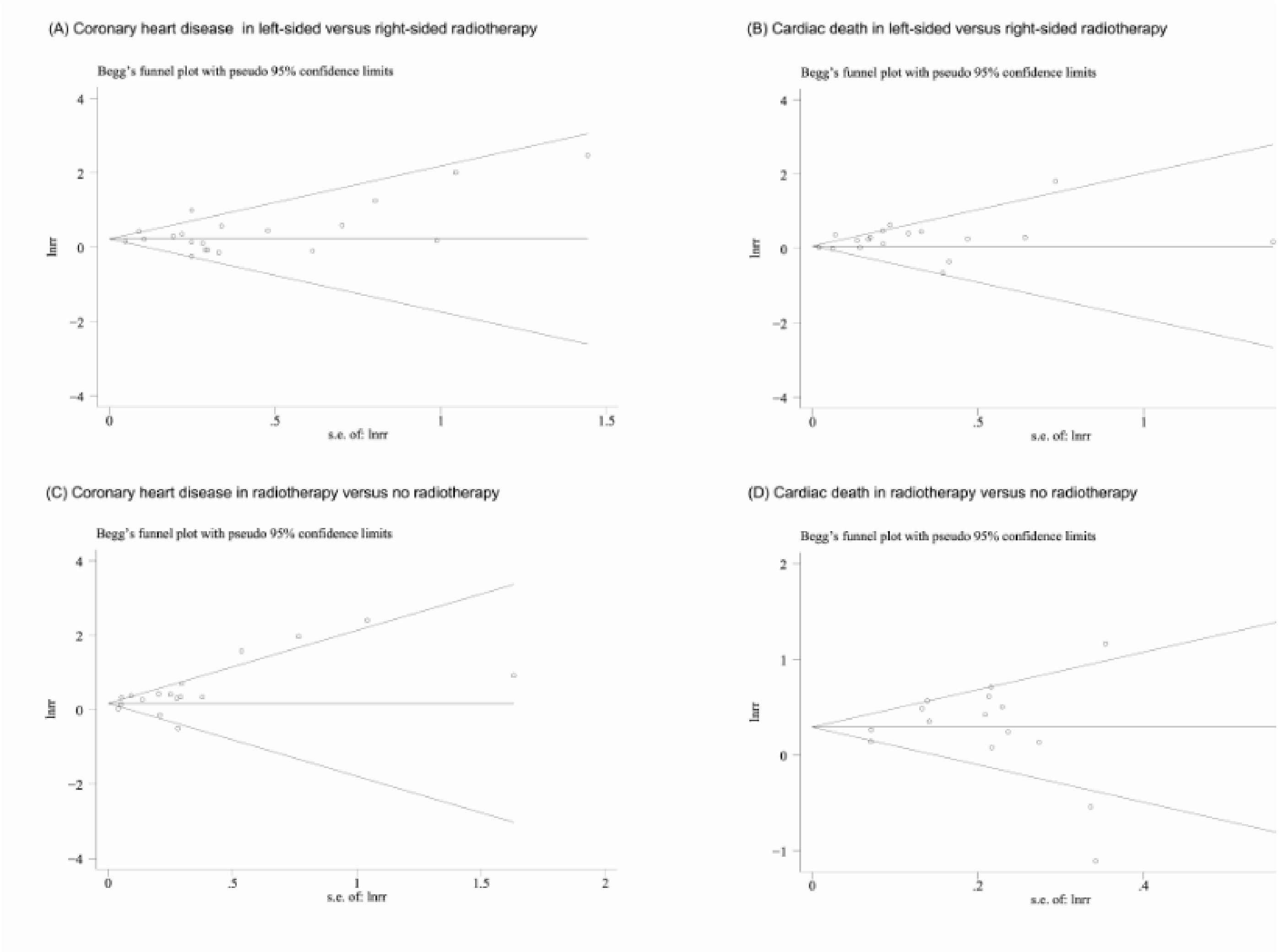

Figure S2. Funnel plots showing association of coronary heart disease and cardiac death with radiotherapy.

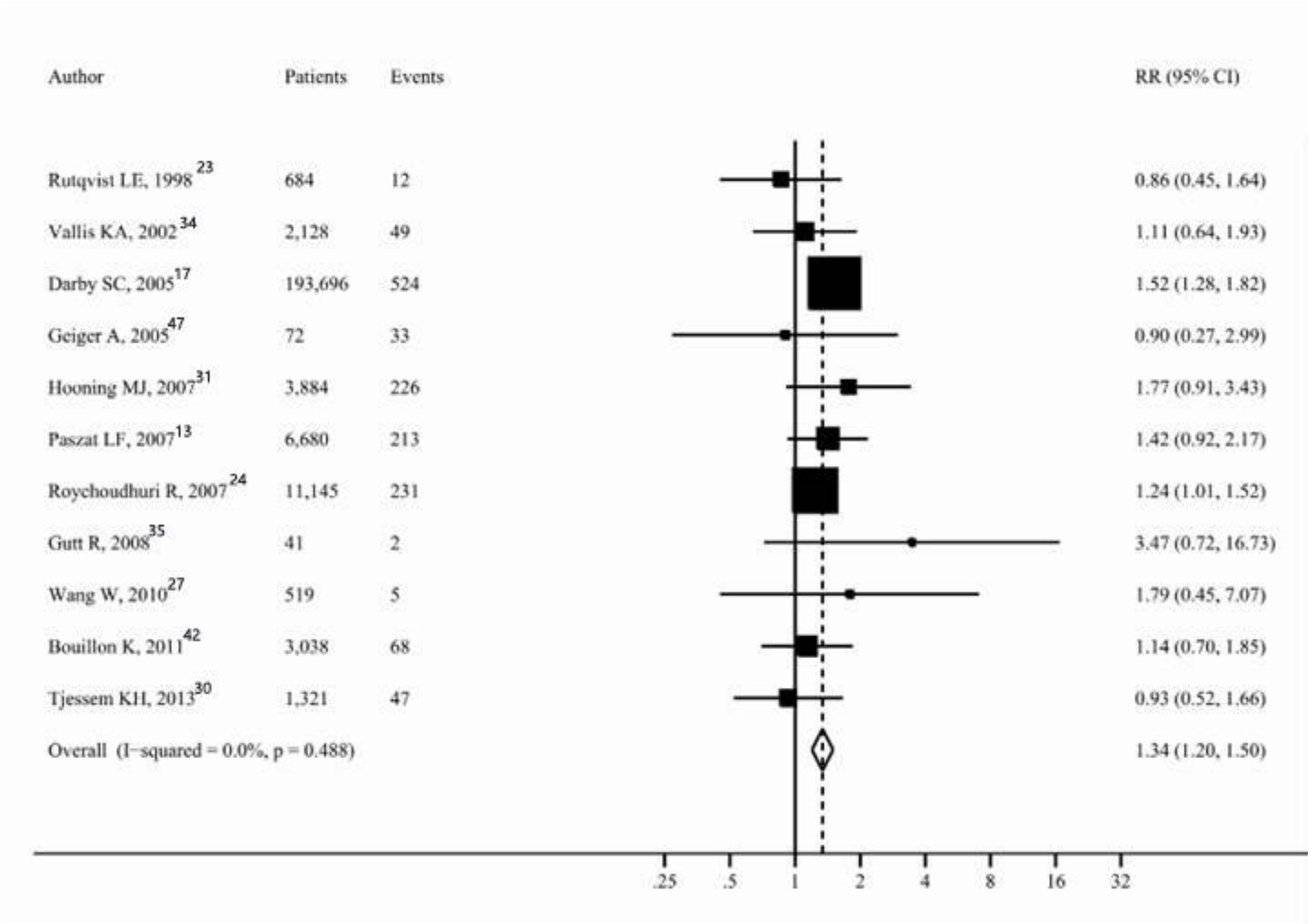

Figure S3. Forest plot for risk of myocardial infarction and death from coronary heart disease in patients with left-sided radiotherapy versus right-sided radiotherapy.

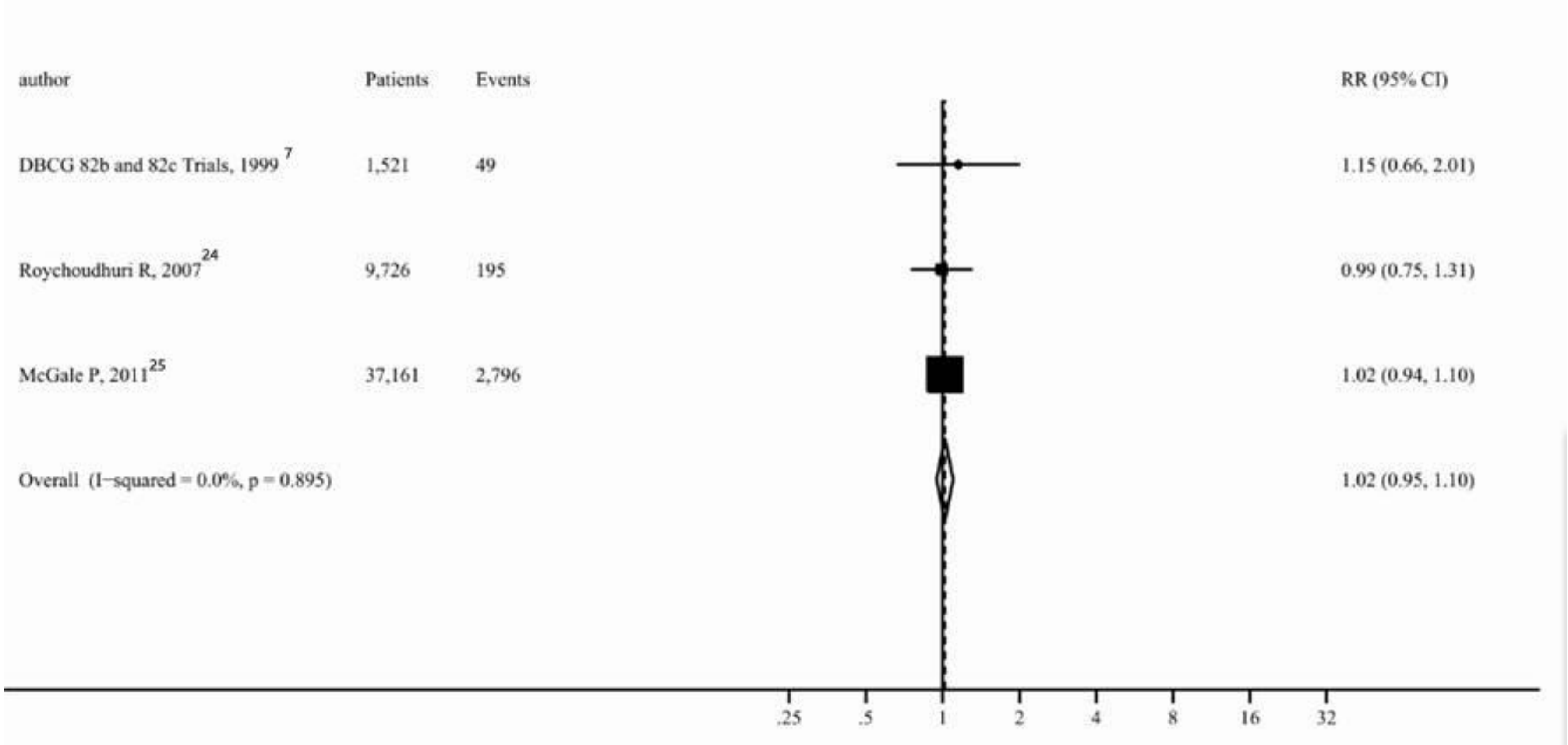

Figure S4. Forest plot for risk of coronary heart disease in unirradiated patients with left-sided versus right-sided breast cancer.

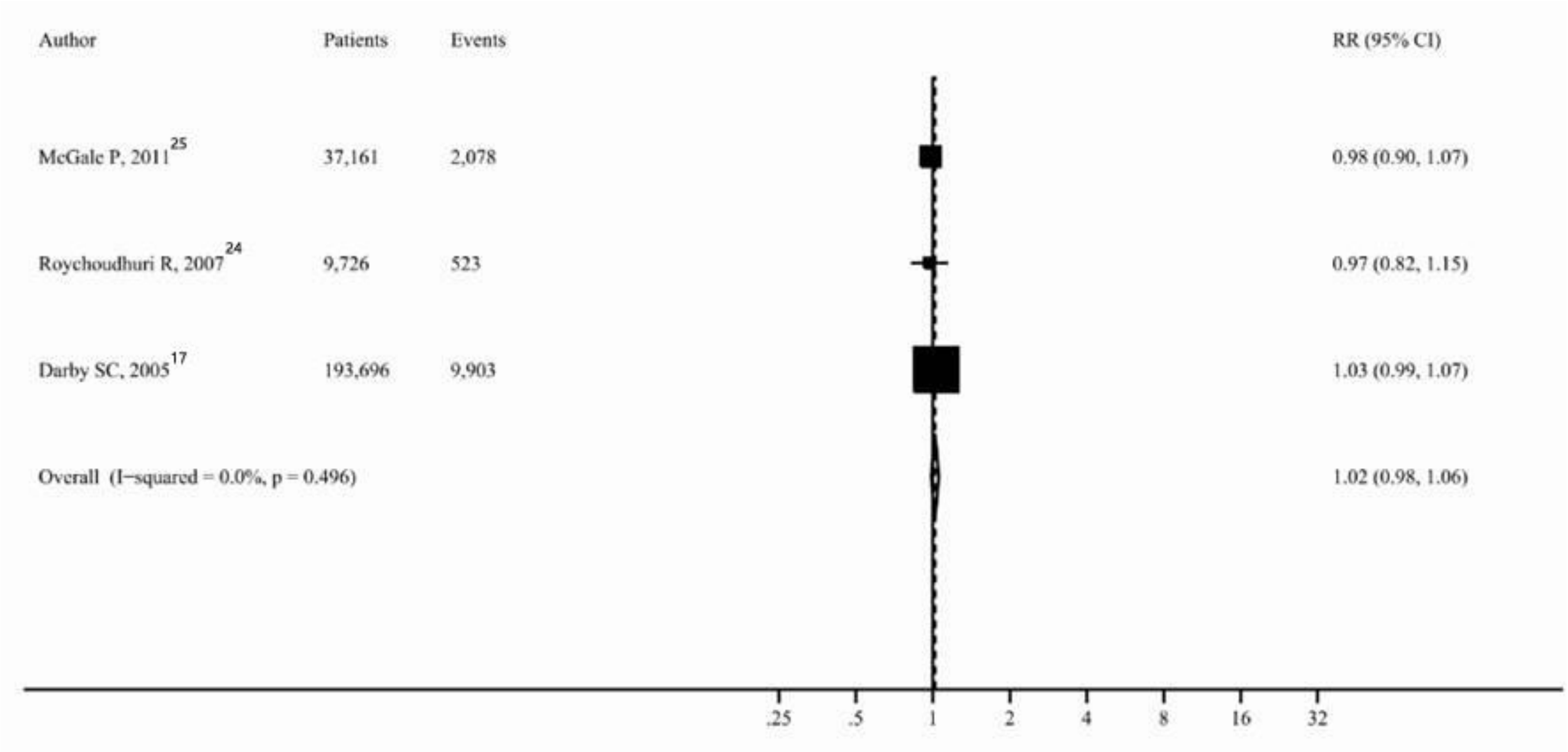

Figure S5. Forest plot for risk of cardiac death in unirradiated patients with left-sided versus right-sided breast cancer.

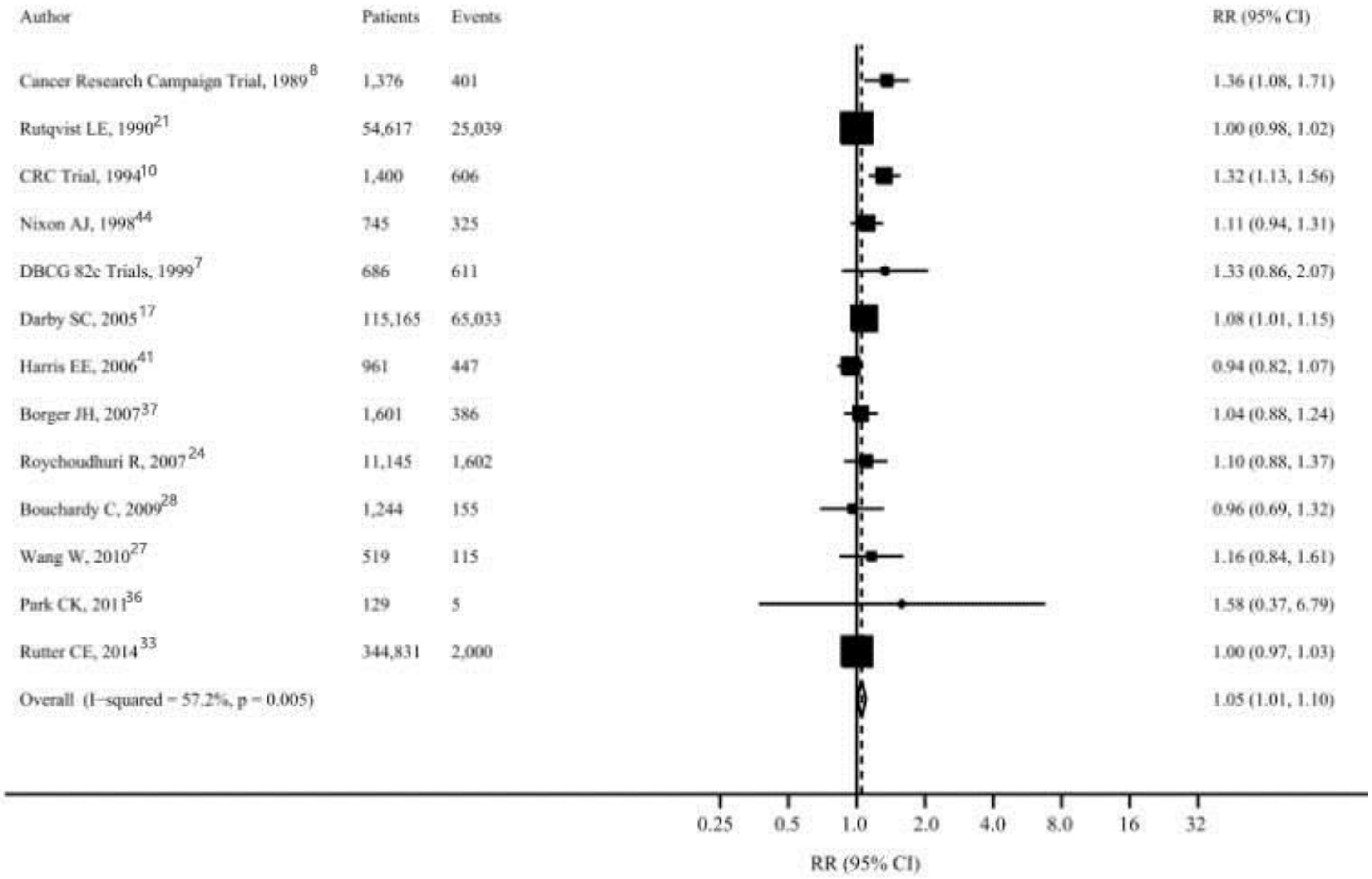

Figure S6. Forest plot for risk of death from any cause in patients with left-sided radiotherapy versus right-sided radiotherapy.

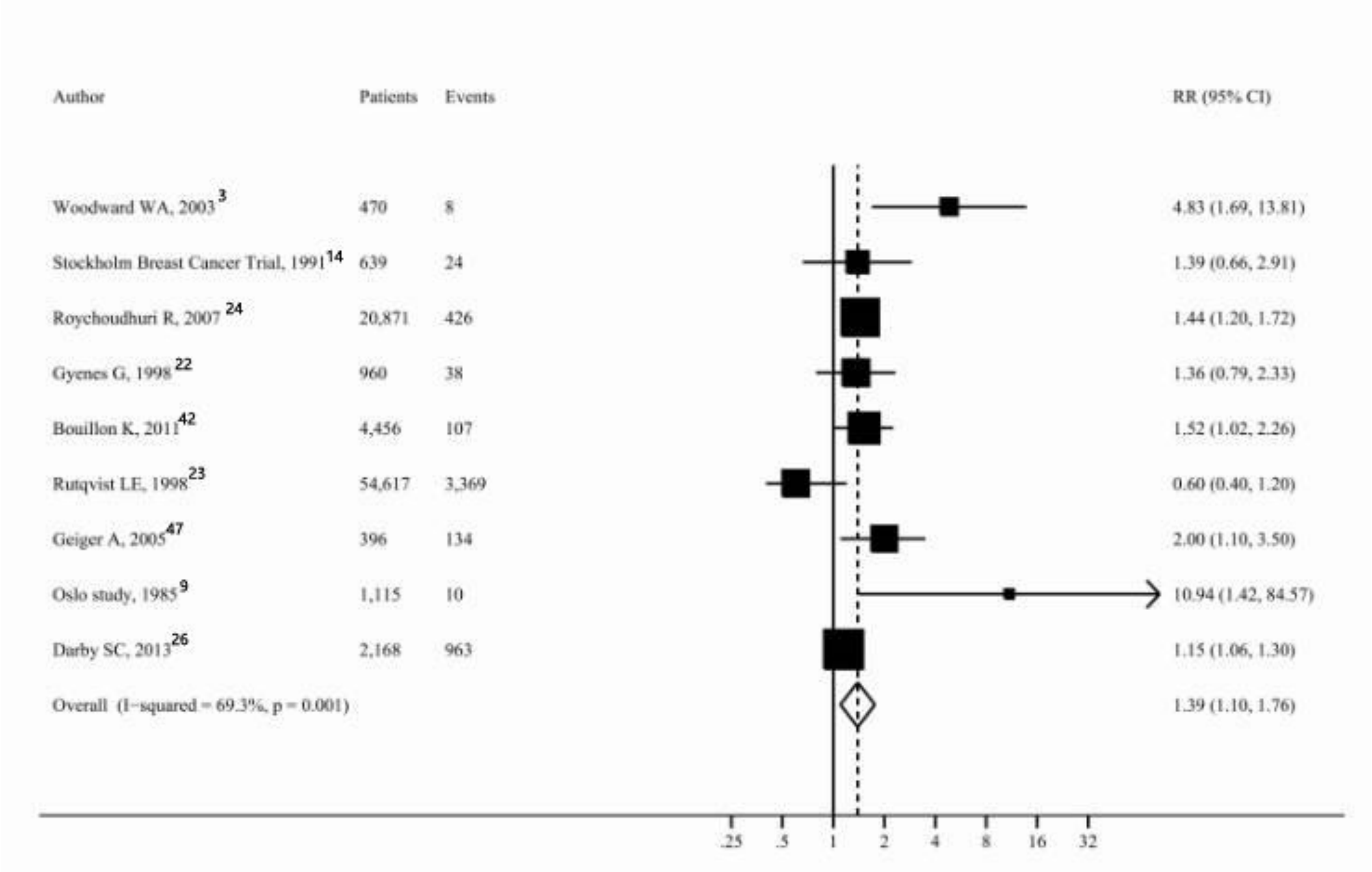

Figure S7. Forest plot for risk of myocardial infarction and death from coronary heart disease in patients with radiotherapy versus without radiotherapy.

Supplemental References:

1. Clarke M, Collins R, Darby S, Davies C, Elphinstone P, Evans V, Godwin J, Gray R, Hicks C, James S, MacKinnon E, McGale P, McHugh T, Peto R, Taylor C, Wang Y. Effects of radiotherapy and of differences in the extent of surgery for early breast cancer on local recurrence and 15-year survival: an overview of the randomised trials. *LANCET.* 2005;366:2087-2106.

2. Valagussa P, Zambetti M, Biasi S, Moliterni A, Zucali R, Bonadonna G. Cardiac effects following adjuvant chemotherapy and breast irradiation in operable breast cancer. *ANN ONCOL.* 1994;5:209-216.

3. Woodward WA, Strom EA, McNeese MD, Perkins GH, Outlaw EL, Hortobagyi GN, Buzdar AU, Buchholz TA. Cardiovascular death and second non-breast cancer malignancy after postmastectomy radiation and doxorubicin-based chemotherapy. *Int J Radiat Oncol Biol Phys.* 2003;57:327-335.

4. Rutqvist LE, Lax I, Fornander T, Johansson H. Cardiovascular mortality in a randomized trial of adjuvant radiation therapy versus surgery alone in primary breast cancer. *Int J Radiat Oncol Biol Phys.* 1992;22:887-896.

5. Cuzick J, Stewart H, Rutqvist L, Houghton J, Edwards R, Redmond C, Peto R, Baum M, Fisher B, Host H, Et A. Cause-specific mortality in long-term survivors of breast cancer who participated in trials of radiotherapy. *J CLIN ONCOL.* 1994;12:447-453.

6. Killander F, Anderson H, Kjellen E, Malmstrom P. Increased cardio and cerebrovascular mortality in breast cancer patients treated with postmastectomy radiotherapy--25 year follow-up of a randomised trial from the South Sweden Breast Cancer Group. *EUR J CANCER.* 2014;50:2201-2210.

7. Hojris I, Overgaard M, Christensen JJ, Overgaard J. Morbidity and mortality of ischaemic heart disease in high-risk breast-cancer patients after adjuvant postmastectomy systemic treatment with or without radiotherapy: analysis of DBCG 82b and 82c randomised trials. Radiotherapy Committee of the Danish Breast Cancer Cooperative Group. *LANCET.* 1999;354:1425-1430.

8. Haybittle JL, Brinkley D, Houghton J, A'Hern RP, Baum M. Postoperative radiotherapy and late mortality: evidence from the Cancer Research Campaign trial for early breast cancer. *BMJ.* 1989;298:1611-1614.

9. Host H, Brennhovd IO, Loeb M. Postoperative radiotherapy in breast cancer--long-term results from the Oslo study. *Int J Radiat Oncol Biol Phys.* 1986;12:727-732.

10. Houghton J, Baum M, Haybittle JL. Role of radiotherapy following total mastectomy in patients with early breast cancer. The Closed Trials Working Party of the CRC Breast Cancer Trials Group. *WORLD J SURG.* 1994;18:117-122.

11. Jones JM, Ribeiro GG. Mortality patterns over 34 years of breast cancer patients in a clinical trial of post-operative radiotherapy. *CLIN RADIOL.* 1989;40:204-208.

12. Halyard MY, Pisansky TM, Dueck AC, Suman V, Pierce L, Solin L, Marks L, Davidson N, Martino S, Kaufman P, Kutteh L, Dakhil SR, Perez EA. Radiotherapy and adjuvant trastuzumab in operable breast cancer: tolerability and adverse event data from the NCCTG Phase III Trial N9831. *J CLIN ONCOL.* 2009;27:2638-2644.

13. Paszat LF, Mackillop WJ, Groome PA, Schulze K, Holowaty E. Mortality from myocardial infarction following postlumpectomy radiotherapy for breast cancer: a population-based study in Ontario, Canada. *Int J Radiat Oncol Biol Phys.* 1999;43:755-762.

14. Patt DA, Goodwin JS, Kuo YF, Freeman JL, Zhang DD, Buchholz TA, Hortobagyi GN, Giordano SH. Cardiac morbidity of adjuvant radiotherapy for breast cancer. *J CLIN ONCOL.* 2005;23:7475-7482.

15. Pinder MC, Duan Z, Goodwin JS, Hortobagyi GN, Giordano SH. Congestive heart failure in older women treated with adjuvant anthracycline chemotherapy for breast cancer. *J CLIN ONCOL.* 2007;25:3808-3815.

16. Harlan LC, Klabunde CN, Ambs AH, Gibson T, Bernstein L, McTiernan A, Meeske K, Baumgartner KB, Ballard-Barbash R. Comorbidities, therapy, and newly diagnosed conditions for women with early stage breast cancer. *J CANCER SURVIV.* 2009;3:89-98.

17. Darby SC, McGale P, Taylor CW, Peto R. Long-term mortality from heart disease and lung cancer after radiotherapy for early breast cancer: prospective cohort study of about 300,000 women in US SEER cancer registries. *LANCET ONCOL.* 2005;6:557-565.

18. Henson KE, McGale P, Taylor C, Darby SC. Radiation-related mortality from heart disease and lung cancer more than 20 years after radiotherapy for breast cancer. *Br J Cancer.* 2013;108:179-182.

19. Doyle JJ, Neugut AI, Jacobson JS, Wang J, McBride R, Grann A, Grann VR, Hershman D. Radiation therapy, cardiac risk factors, and cardiac toxicity in early-stage breast cancer patients. *Int J Radiat Oncol Biol Phys.* 2007;68:82-93.

20. Giordano SH, Kuo YF, Freeman JL, Buchholz TA, Hortobagyi GN, Goodwin JS. Risk of cardiac death after adjuvant radiotherapy for breast cancer. *J Natl Cancer Inst.* 2005;97:419-424.

21. Rutqvist LE, Johansson H. Mortality by laterality of the primary tumour among 55,000 breast cancer patients from the Swedish Cancer Registry. *Br J Cancer.* 1990;61:866-868.

22. Gyenes G, Rutqvist LE, Liedberg A, Fornander T. Long-term cardiac morbidity and mortality in a randomized trial of pre- and postoperative radiation therapy versus surgery alone in primary breast cancer. *RADIOTHER ONCOL.* 1998;48:185-190.

23. Rutqvist LE, Liedberg A, Hammar N, Dalberg K. Myocardial infarction among women with

early-stage breast cancer treated with conservative surgery and breast irradiation. *Int J Radiat Oncol Biol Phys.* 1998;40:359-363.

24. Roychoudhuri R, Robinson D, Putcha V, Cuzick J, Darby S, Moller H. Increased cardiovascular mortality more than fifteen years after radiotherapy for breast cancer: a population-based study. *BMC CANCER.* 2007;7:9.

25. McGale P, Darby SC, Hall P, Adolfsson J, Bengtsson NO, Bennet AM, Fornander T, Gigante B, Jensen MB, Peto R, Rahimi K, Taylor CW, Ewertz M. Incidence of heart disease in 35,000 women treated with radiotherapy for breast cancer in Denmark and Sweden. *RADIOTHER ONCOL.* 2011;100:167-175.

26. Darby SC, Ewertz M, McGale P, Bennet AM, Blom-Goldman U, Bronnum D, Correa C, Cutter D, Gagliardi G, Gigante B, Jensen MB, Nisbet A, Peto R, Rahimi K, Taylor C, Hall P. Risk of ischemic heart disease in women after radiotherapy for breast cancer. *N Engl J Med.* 2013;368:987-998.

27. Wang W, O'Connell D, Stuart K, Boyages J. Analysis of 10-year cause-specific mortality of patients with breast cancer treated in New South Wales in 1995. *J Med Imaging Radiat Oncol.* 2011;55:516-525.

28. Bouchardy C, Rapiti E, Usel M, Majno SB, Vlastos G, Benhamou S, Miralbell R, Neyroud-Caspar I, Verkooijen HM, Vinh-Hung V. Excess of cardiovascular mortality among node-negative breast cancer patients irradiated for inner-quadrant tumors. *ANN ONCOL.* 2010;21:459-465.

29. Stokes EL, Tyldesley S, Woods R, Wai E, Olivotto IA. Effect of nodal irradiation and fraction size on cardiac and cerebrovascular mortality in women with breast cancer treated with local and locoregional radiotherapy. *Int J Radiat Oncol Biol Phys.* 2011;80:403-409.

30. Tjessem KH, Johansen S, Malinen E, Reinertsen KV, Danielsen T, Fossa SD, Fossa A. Long-term cardiac mortality after hypofractionated radiation therapy in breast cancer. *Int J Radiat Oncol Biol Phys.* 2013;87:337-343.

31. Hooning MJ, Botma A, Aleman BM, Baaijens MH, Bartelink H, Klijn JG, Taylor CW, van Leeuwen FE. Long-term risk of cardiovascular disease in 10-year survivors of breast cancer. *J Natl Cancer Inst.* 2007;99:365-375.

32. Boekel NB, Schaapveld M, Gietema JA, Rutgers EJ, Versteegh MI, Visser O, Aleman BM, van Leeuwen FE. Cardiovascular morbidity and mortality after treatment for ductal carcinoma in situ of the breast. *J Natl Cancer Inst.* 2014;106.

33. Rutter CE, Chagpar AB, Evans SB. Breast cancer laterality does not influence survival in a large modern cohort: implications for radiation-related cardiac mortality. *Int J Radiat Oncol Biol Phys.* 2014;90:329-334.

34. Vallis KA, Pintilie M, Chong N, Holowaty E, Douglas PS, Kirkbride P, Wielgosz A. Assessment of coronary heart disease morbidity and mortality after radiation therapy for early breast cancer. *J CLIN ONCOL.* 2002;20:1036-1042.

35. Gutt R, Correa CR, Hwang WT, Solin LJ, Litt HI, Ferrari VA, Harris EE. Cardiac morbidity and mortality after breast conservation treatment in patients with early-stage breast cancer and preexisting cardiac disease. *CLIN BREAST CANCER.* 2008;8:443-448.

36. Park CK, Li X, Starr J, Harris EE. Cardiac morbidity and mortality in women with ductal carcinoma in situ of the breast treated with breast conservation therapy. *BREAST J.* 2011;17:470-476.

37. Borger JH, Hooning MJ, Boersma LJ, Snijders-Keilholz A, Aleman BM, Lintzen E, van Brussel S, van der Toorn PP, Alwhouhayb M, van Leeuwen FE. Cardiotoxic effects of tangential breast irradiation in early breast cancer patients: the role of irradiated heart volume. *Int J Radiat Oncol Biol Phys.* 2007;69:1131-1138.

38. Dubois CL, Pappas C, Belmans A, Erven K, Adriaenssens T, Sinnaeve P, Coosemans M, Kayaert P, Weltens C, Desmet W. Clinical outcome of coronary stenting after thoracic radiotherapy: a case-control study. *HEART.* 2010;96:678-682.

39. Correa CR, Litt HI, Hwang WT, Ferrari VA, Solin LJ, Harris EE. Coronary artery findings after left-sided compared with right-sided radiation treatment for early-stage breast cancer. *J CLIN ONCOL.* 2007;25:3031-3037.

40. Hooning MJ, Aleman BM, van Rosmalen AJ, Kuenen MA, Klijn JG, van Leeuwen FE. Cause-specific mortality in long-term survivors of breast cancer: A 25-year follow-up study. *Int J Radiat Oncol Biol Phys.* 2006;64:1081-1091.

41. Harris EE, Correa C, Hwang WT, Liao J, Litt HI, Ferrari VA, Solin LJ. Late cardiac mortality and morbidity in early-stage breast cancer patients after breast-conservation treatment. *J CLIN ONCOL.* 2006;24:4100-4106.

42. Bouillon K, Haddy N, Delaloge S, Garbay JR, Garsi JP, Brindel P, Mousannif A, Le MG, Labbe M, Arriagada R, Jougla E, Chavaudra J, Diallo I, Rubino C, de Vathaire F. Long-term cardiovascular mortality after radiotherapy for breast cancer. *J AM COLL CARDIOL.* 2011;57:445-452.

43. Boerman LM, Berendsen AJ, van der Meer P, Maduro JH, Berger MY, de Bock GH. Long-term follow-up for cardiovascular disease after chemotherapy and/or radiotherapy for breast cancer in an unselected population. *SUPPORT CARE CANCER.* 2014;22:1949-1958.

44. Nixon AJ, Manola J, Gelman R, Bornstein B, Abner A, Hetelekidis S, Recht A, Harris JR. No long-term increase in cardiac-related mortality after breast-conserving surgery and radiation therapy using modern techniques. *J CLIN ONCOL.* 1998;16:1374-1379.

45. Jagsi R, Griffith KA, Koelling T, Roberts R, Pierce LJ. Rates of myocardial infarction and coronary artery disease and risk factors in patients treated with radiation therapy for early-stage breast cancer. *CANCER-AM CANCER SOC.* 2007;109:650-657.

46. Caussa L, Kirova YM, Gault N, Pierga JY, Savignoni A, Campana F, Dendale R, Fourquet A, Bollet MA. The acute skin and heart toxicity of a concurrent association of trastuzumab and locoregional breast radiotherapy including internal mammary chain: a single-institution study. *EUR J CANCER.* 2011;47:65-73.

47. Geiger AM, Chen W, Bernstein L. Myocardial infarction risk and tamoxifen therapy for breast cancer. *Br J Cancer.* 2005;92:1614-1620.
